# Inferring population structure in biobank-scale genomic data

**DOI:** 10.1016/j.ajhg.2022.02.015

**Published:** 2022-03-16

**Authors:** Alec M. Chiu, Erin K. Molloy, Zilong Tan, Ameet Talwalkar, Sriram Sankararaman

**Affiliations:** 1 Bioinformatics Interdepartmental Program, University of California, Los Angeles, Los Angeles, CA 90095, USA; 2 Department of Computer Science, University of California, Los Angeles, Los Angeles, CA 90095, USA; 3 Institute for Advanced Computer Studies, University of Maryland, College Park, College Park, MD 20742, USA; 4 Facebook, Inc., Menlo Park, CA 94025, USA; 5 Machine Learning Department, Carnegie Mellon University, Pittsburgh, PA 15213, USA; 6 Department of Human Genetics, University of California, Los Angeles, Los Angeles, CA 90095, USA; 7 Department of Computational Medicine, David Geffen School of Medicine, University of California, Los Angeles, Los Angeles, CA 90095, USA

**Keywords:** population structure, admixture, genetic structure, biobank, scalability, ancestry

## Abstract

Inferring the structure of human populations from genetic variation data is a key task in population and medical genomic studies. Although a number of methods for population structure inference have been proposed, current methods are impractical to run on biobank-scale genomic datasets containing millions of individuals and genetic variants. We introduce SCOPE, a method for population structure inference that is orders of magnitude faster than existing methods while achieving comparable accuracy. SCOPE infers population structure in about a day on a dataset containing one million individuals and variants as well as on the UK Biobank dataset containing 488,363 individuals and 569,346 variants. Furthermore, SCOPE can leverage allele frequencies from previous studies to improve the interpretability of population structure estimates.

## Introduction

Inference of population structure is a central problem in human genetics with applications that range from fine-grained understanding of human history^1^ to correcting for population stratification in genome-wide association studies (GWASs).^2^ Approaches to population structure inference^3^, ^4^, ^5^, ^6^, ^7^, ^8^ typically formalize the problem as one of estimating admixture proportions of each individual and ancestral population allele frequencies given genetic variation data.

The growth of repositories of genetic variation data over large numbers of individuals has opened up the possibility of inferring population structure at increasingly finer resolution.^9^^,^^10^ For instance, the UK Biobank^9^ contains genotype data from approximately half a million British individuals across millions of SNPs. This development has necessitated methods that can be applied to large-scale datasets with reasonable runtime and memory requirements. Existing methods, however, do not scale to these datasets. Thus, we have developed SCOPE (scalable population structure inference)—a scalable method capable of inferring population structure on biobank-scale data.

SCOPE utilizes a previously proposed likelihood-free framework^8^ that involves estimation of the individual allele frequency (IAF) matrix through a statistical technique known as latent subspace estimation (LSE)^11^ followed by a decomposition of the estimated IAF matrix into ancestral allele frequencies and admixture proportions. SCOPE uses two ideas to substantially improve the scalability of this approach. First, SCOPE uses randomized eigendecomposition^12^ to efficiently estimate the latent subspace. Specifically, SCOPE avoids the need to form matrices that are expensive to compute on or require substantial memory and instead works directly with the input genotype matrix. Second, SCOPE leverages the insight that the resulting method involves repeated multiplications of the genotype matrix and uses the Mailman algorithm for fast multiplication of the genotype matrix.^13^

We benchmarked the accuracy and efficiency of SCOPE on simulated and real datasets. In simulations, SCOPE obtains accuracy comparable to existing methods while being up to 1,800 times faster. Relative to the previous state-of-the-art scalable method (TeraStructure^7^), SCOPE is three to 144 times faster. SCOPE can estimate population structure in about a day for a simulated dataset consisting of one million individuals and SNPs for six latent populations, whereas TeraStructure is extrapolated to require approximately 20 days on this same dataset. We additionally used SCOPE to infer continental ancestry proportions (four ancestry groups) on the UK Biobank dataset (488,363 individuals and 569,346 SNPs) in about a day. We find that the inferred continental ancestry proportions are highly concordant with self-reported race and ethnicity (SIRE).

SCOPE additionally can be applied in a supervised setting. Given allele frequencies from reference populations,^14^^,^^15^ SCOPE can estimate admixture proportions corresponding to the reference populations to enable greater interpretability.

## Subjects and methods

### The structure/admixture model

The structure/admixture model links the m×n genotype matrix ***X*** (where rows refer to single nucleotide polymorphisms [SNPs] and columns refer to individual diploid genotypes, $x_{ij}\;\in \;\left\{0,1,2\right\},i\;\in \;\left\{1,\ldots ,m\right\},j\;\in \;\left\{1,\ldots ,n\right\}$) to the m×n individual allele frequency (IAF) matrix ***F***, m×k ancestral population allele frequencies ***P***, and the k×n individual admixture proportions ***Q*** (also termed the global ancestry of an individual). Here, *m* denotes the number of SNPs, *n* denotes the number of individuals, and *k* denotes the number of latent populations. The IAF matrix, ancestral allele frequencies, and admixture proportions are mathematically related as F=PQ. Furthermore, there are constraints on ***P*** and ***Q***. Each element of ***P*** is constrained to lie between 0 and 1 $\left(0\leq p_{il}\leq 1,i\;\in \;\left\{1,\ldots ,m\right\},l\;\in \;\left\{1,\ldots ,k\right\}\right)$. Each element of ***Q*** is non-negative $\left(q_{lj}\geq 0,l\;\in \;\left\{1,\ldots ,k\right\},j\;\in \;\left\{1,\ldots ,n\right\}\right)$ and the admixture proportion of each individual must sum to one $\left(\sum_{l}q_{lj}=1\right)$. Finally, each entry of the genotype matrix is an independent draw from the corresponding entry of the IAF matrix *F* as: $x_{ij}|f_{ij}∼\text{Binomial}\left(2,f_{ij}\right)$. The goal of population structure inference under the structure/admixture model is to estimate ***P*** and ***Q*** given ***X***.

### SCOPE

For scalable inference, SCOPE uses as its starting point a likelihood-free estimator of population structure previously proposed in ALStructure.^8^ This estimator has two major steps: latent subspace estimation (LSE) and alternating least-squares (ALS). LSE attempts to estimate the subspace spanned by the rows of ***Q***^11^ by computing a low-rank approximation to the matrix $G=\left(1/m\right)X^{T}X-D$ where each entry $d_{j}$ of the n×n diagonal matrix ***D*** is obtained as $d_{j}=\left(1/m\right)\sum_{i=1}^{m}2x_{ij}-x_{ij}^{2}$. The latent subspace of ***Q*** is estimated as the span of the top *k* eigenvectors of ***G*:** $v_{1},\ldots ,v_{k}$. After obtaining the top *k* eigenvectors $V=\left[v_{1},\ldots ,v_{k}\right]$, ALStructure projects the data ***X*** onto ***V*** to obtain an estimate of ***F*:** $\hat{F}=\left(1/2\right)\mathrm{XV}V^{T}$. It then uses truncated alternating least-squares (ALS) to factorize the estimate, $\hat{F}$, into estimates of ***P*** and Q: $\hat{F}=\hat{P}\hat{Q}$. $\hat{Q}$ are the estimates of the individual admixture proportions.

A naive approach to compute the top *k* eigenvectors of *G* would involve first forming the matrix ***G*** and then computing its top *k* eigenvectors, which would require $O(n^{2}m+n^{2}k)$ (if a full SVD is performed, this step would require $O(\text{min}\left(n,m\right)nm)$). To perform scalable LSE, SCOPE uses techniques from randomized linear algebra,^12^ specifically the implicitly restarted Arnoldi method,^16^ to obtain the top *k* eigenvectors. This step involves repeatedly multiplying estimates of the eigenvectors $v_{l}:l\;\in \;\left\{1,\ldots ,k\right\}$ with the genotype matrix: $\left(\left(1/m\right)X^{T}X-D\right)v_{l}=\left(1/m\right){\left({\left(Xv_{l}\right)}^{T}X\right)}^{T}-Dv_{l}$ and can be performed without explicitly forming the matrix ***G***. Instead, this approach requires repeatedly computing $w_{l}\equiv Xv_{l}$, $w_{l}^{T}X$, and $Dv_{l}$, which can be computed in O(nmk) time. We use the C++ Spectra library (web resources) to implement these computations in SCOPE.

To efficiently compute $\hat{P}$ and $\hat{Q}$ with truncated ALS, we randomly initialized the matrix $\hat{P}$ with all values between 0 and 1 $\left(0\leq {\hat{p}}_{il}\leq 1\right)$. We iteratively solve for estimates of ***P*** and ***Q***, projecting the estimates onto the constraint space until convergence:

$$\hat{Q}=\frac{1}{2}{\left({\hat{P}}^{T}\hat{P}\right)}^{-1}{\hat{P}}^{T}\mathrm{XV}V^{T}$$

$$\hat{P}=\frac{1}{2}\mathrm{XV}V^{T}\hat{Q}{\left(\hat{Q}{\hat{Q}}^{T}\right)}^{-1}.$$

All values in $\hat{P}$ are truncated to be between 0 and 1 while $\hat{Q}$ is projected onto the appropriate simplex. Each step of the ALS algorithm has runtime O(nmk). We note here that we never store $\hat{F}$ but instead compute it implicitly per iteration. This allows us to reduce the memory footprint of SCOPE, as $\hat{F}$ is a continuous, real-valued matrix with the same dimensions as the genotype matrix. It is not feasible for most computers to be able to store this in memory. For instance, to store our larger UK Biobank dataset (488,363 individuals and 569,346 SNPs), one is estimated to require around 2,072 GB of memory.

Each of the computations in SCOPE requires multiplying a genotype matrix with entries consisting of only 0, 1, and 2 for diploid genotype. These operations can be efficiently performed with the Mailman algorithm,^13^ which provides computational savings when there are repeated multiplications involving a matrix with a finite alphabet. We utilize the Mailman algorithm in computations involving the genotype matrix in both LSE and ALS so that the final time complexity of SCOPE is $O\left(nmk/\text{max}\left({\text{log}}_{3}n,{\text{log}}_{3}m\right)\right)$.

### Supervised population structure inference

SCOPE can utilize allele frequencies from reference populations to infer corresponding admixture proportions. In this scenario, we assume $\hat{P}$, the population allele frequencies, are known. As a result, one only needs to compute $\hat{Q}$ by using the supplied $\hat{P}$. This allows the admixture proportions corresponding to the reference populations to be inferred in a single step of ALS once the LSE step is completed.

### Permutation matching of inferred results

The output of population structure inference methods can result in output that is permuted even between different runs of the same method. It is critical to correctly match latent populations between methods and runs in order to properly assess results. To perform permutation matching, we employed a strategy similar to that of Behr et al.^17^ This permutation matching problem is better known as the assignment problem, which can be solved efficiently with linear programming. We first construct a score matrix by using the distance metric created in Behr et al.^17^ The optimal permutation match can then be found by optimizing the total score from assignments through linear programming. We utilize the *lpSolve* (web resources) package in R to solve the linear program.

### PSD model simulations

We perform simulations under the STRUCTURE or Pritchard-Stephens-Donnelly (PSD) model.^3^ In the PSD model, priors are placed on ***P*** and ***Q***:

$$p_{il}\;\overset{iid}{∼}\;\text{Beta}\left(\frac{1-F_{ST}}{F_{ST}}p_{A},\frac{1-F_{ST}}{F_{ST}}\left(1-p_{A}\right)\right),i\;\in \;\left\{1,\ldots ,m\right\},l\;\in \;\left\{1,\ldots ,k\right\}$$

$$q_{:,j}\;\overset{iid}{∼}\;\text{Dirichlet}\;\left(\alpha 1_{K}\right),j\;\in \;\left\{1,\ldots ,n\right\}.$$

The allele frequencies $p_{il}$ are drawn from the Balding-Nichols model,^18^ which is a beta distribution parametrized by the fixation index $\left(F_{ST}\right)$ and an initial allele frequency $\left(p_{A}\right)$. For our simulations, we calculated $F_{ST}$ and $p_{A}$ from our real datasets. Admixture proportions $q_{:,j}$ are drawn at random from a Dirichlet distribution. We take the product of the two matrices to form the IAF matrix, F=PQ, and draw each genotype from a binomial distribution parametrized by entries of ***F*:** $x_{ij}∼\text{Binomial}\left(2,f_{ij}\right)$.

### Spatial model simulations

We also perform simulations under a spatial model similar to that in Ochoa and Storey.^19^ In the spatial model, allele frequencies $p_{il}$ are drawn as in the PSD model, but the admixture proportions, ***q***, are drawn from a 1D geography.

z≡(1,…,k)

$$y_{j}\;\overset{iid}{∼}\;\text{Uniform}\left(0,k+1\right)$$

$$q_{lj}=\frac{f_{z_{l}}\left(y_{j}\right)}{\sum_{l=1}^{k}f_{z_{l}}\left(y_{j}\right)}$$

Populations are placed at integer values on a line. We get the resulting population position vector, z≡(1,…,k). Each individual has a position, $y_{j}$ drawn from a uniform distribution between 0 and k+1. Proportions for each population are generated via a normal distribution, where $f_{z_{l}}$ denotes the normal density function with $z_{l}$ (l∈{1,…,k}) as the mean and $\sigma^{2}$ as variance. The resulting vector of proportions is then normalized to satisfy the constraints on ***Q***. We used $\sigma^{2}=4$ for our simulations.

### Assessment of results

We assess our results by using two metrics: average Jensen-Shannon divergence (JSD) and average root-mean-square error (RMSE). We calculate the metrics between the true global ancestry proportions, ***Q***, and the estimates, $\hat{Q}$, after $\hat{Q}$ has been permutation matched to the true proportions.

$$\text{RMSE}\left(Q,\hat{Q}\right)=\frac{1}{\sqrt{nk}}{\left\|Q-\hat{Q}\right\|}_{F}$$

$$\text{JSD}\left(Q,\hat{Q}\right)=\frac{1}{2}\left[\text{KL}\left(Q,\frac{1}{2}\left[Q+\hat{Q}\right]\right)+\text{KL}\left(\hat{Q},\frac{1}{2}\left[Q+\hat{Q}\right]\right)\right]$$

${\left\|\cdot \right\|}_{F}$ represents the Frobenius norm. KL is the Kullback-Leibler divergence, which is defined as:

$$\text{KL}\left(Q,\hat{Q}\right)=\frac{1}{n}\sum_{j=1}^{n}\sum_{l=1}^{k}q_{lj}\text{log}\left(\frac{q_{lj}}{{\hat{q}}_{lj}}\right).$$

In the JSD calculations, we replace values of 0 in ***Q*** or $\hat{Q}$ with $1\times 10^{-9}$ to avoid numerical issues.

### Datasets

We use the 1000 Genomes Project (TGP),^14^^,^^15^ Human Origins (HO),^20^ Human Genome Diversity Project (HGDP),^21^^,^^22^ and the UK Biobank (UKB)^9^ in this study. The HGDP dataset is the complete Stanford HGDP SNP genotyping data filtered to only include individuals in the H952 set,^23^ greater than 95% genotyping rate, and greater than 1% minor allele frequency (MAF), resulting in 940 individuals and 642,951 SNPs. The TGP dataset is the 2012-01-31 Omni Platform genotypes filtered to only include unrelated individuals, greater than 95% genotyping rate, and greater than 1% MAF, resulting in 1,718 individuals and 1,854,622 SNPs. The HO dataset was filtered for human-only samples, greater than 99% genotyping rate, and greater than 5% MAF, resulting in 1,931 individuals and 385,089 SNPs. For the UK Biobank, we filtered the UK Biobank Axiom Array genotypes for greater than 1% MAF, long-range linkage disequilibrium (LD), and pairwise LD pruning in 50 kilobase windows, 80 variant step size, and an $r^{2}$ threshold of 0.1, resulting in 488,363 individuals and 568,346 SNPs. This is similar to the UK Biobank manuscript’s first round of quality control for principal-component analysis (PCA)^9^ with the differences of using all individuals and no genotype filter. We also use the UK Biobank’s final set of PCA SNPs,^9^ which consists of 147,604 SNPs, to explore higher number of latent populations. We calculate metrics such as $F_{ST}$ from the provided population and superpopulation labels provided by each dataset. To perform our supervised analyses, we use the common SNPs between the datasets involved. All genotype processing was performed with PLINK.^24^ Links to the publicly available datasets as well as scripts to apply our preprocessing are available in the code repository for SCOPE.

### Visualization of results

We visualize our inferred admixture proportions as stacked bar plots. We permutation matched estimates from all methods to enable easy comparison. For our PSD simulations, we performed hierarchical clustering with complete linkage on a Euclidean distance matrix calculated from the true admixture proportion matrix (***Q***) to obtain the order of samples. For our spatial simulations, we sorted by decreasing membership of the first population. For our real datasets, we perform the same hierarchical clustering strategy used for our PSD simulations but use the estimates from ADMIXTURE $\left(\hat{Q}\right)$ in place of the true admixture proportions. For the HGDP, TGP, and UK Biobank, we first took the average proportions for each SIRE group and performed hierarchical clustering on the averages to determine the order of the SIRE groups. We then performed hierarchical clustering within each SIRE group to determine the order of individuals within groups. For large datasets, we utilized *genieclust*,^25^ a scalable method for hierarchical clustering.

### Benchmarking

We compared SCOPE to ADMIXTURE v1.3.0,^5^ fastSTRUCTURE,^6^ TeraStructure,^7^ ALStructure v0.1.0,^8^ and sNMF v1.2.^26^

ADMIXTURE computes maximum-likelihood estimates while TeraStructure and fastSTRUCTURE compute approximate posterior estimates in a Bayesian model with variational inference. ALStructure, the framework that SCOPE builds upon, utilizes a two-stage strategy of first performing dimensionality reduction (latent subspace estimation) followed by matrix factorization (alternating least-squares).

Each method was run with eight threads with the exception of fastSTRUCTURE and ALStructure, which do not have multi-threaded implementations. Default parameters were used. TeraStructure has an additional “rfreq” parameter, which was set to 10% of the number of SNPs as recommended by its authors. For SCOPE, we used convergence criteria of either 1,000 iterations of the ALS algorithm or a change between iterations less than $1\times 10^{-5}$, which we calculate as the RMSE between the estimated admixture matrices between two iterations. All experiments were performed on a server with two AMD EPYC 7501 32-Core Processors and 1 terabyte of RAM.

## Results

### Accuracy

We assessed the accuracy of SCOPE by using simulations under the Pritchard-Stephens-Donnelly (PSD) model^3^ to study accuracy under a standard population genetics model and a basic model of spatial structure^19^ to study the robustness of SCOPE and other methods in the presence of model violations. We simulated several independent datasets by using parameters calculated from two real datasets: the 1000 Genomes Project (TGP)^15^ and the Human Genome Diversity Project (HGDP)^27^ (see “benchmarking” sections of subjects and methods). It is important to note that each simulation dataset was created independently of the others and they are not subsets of the largest dataset. Thus, performance should only be compared between methods run on the same dataset.

**Figure 1:**
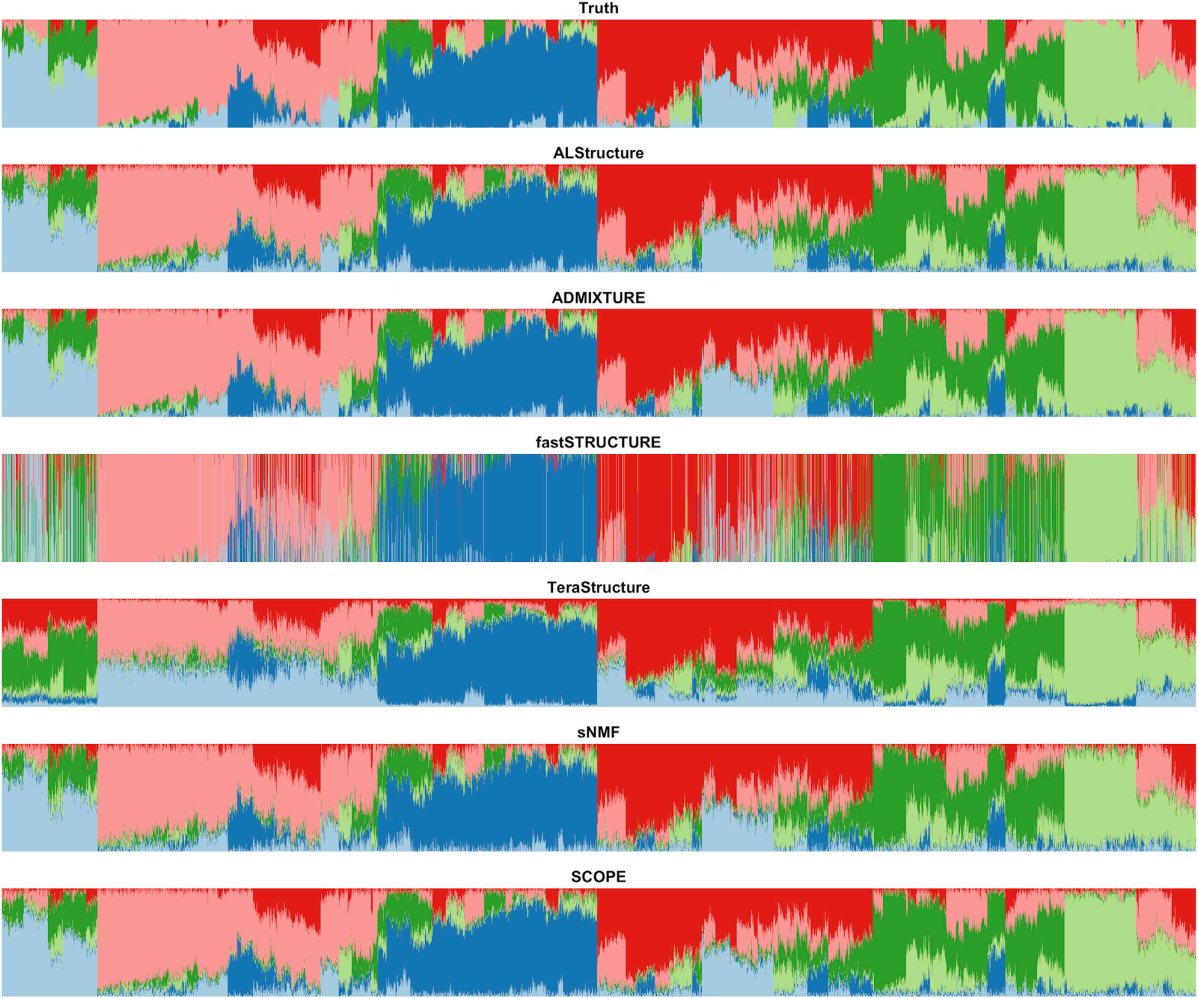
Population structure inference for simulations under PSD model generated with 1000 Genomes Phase 3 data PSD model parameters were drawn from TGP data to generate a simulation dataset with 10,000 samples and 10,000 SNPs. The true admixture proportions and resulting inferred admixture proportions from each method are shown. Colors and order of samples are matched between each method to the truth.

**Figure 2:**
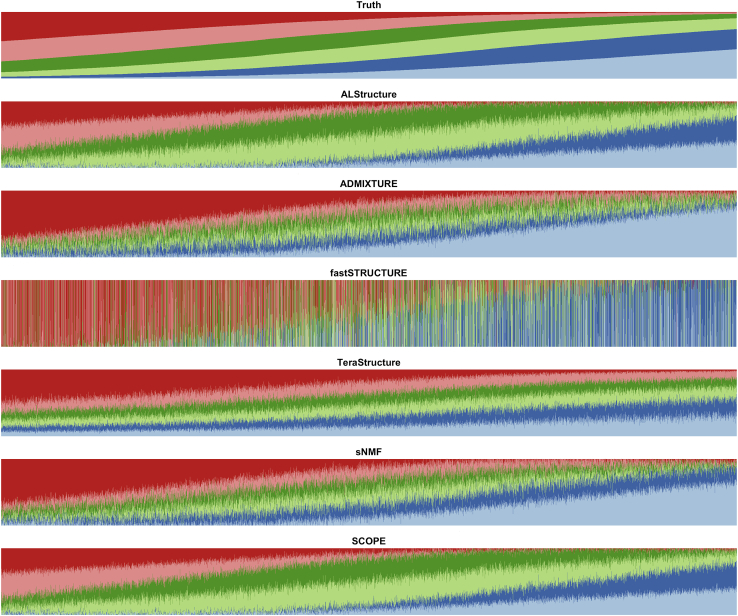
Population structure inference for simulations under a spatial model generated with 1000 Genomes Phase 3 data Model parameters were drawn from TGP data to generate a simulation dataset with 10,000 samples and 10,000 SNPs under a spatial model (see subjects and methods). The true admixture proportions and resulting inferred admixture proportions from each method are shown. Colors and order of samples are matched between each method to the truth.

Under the PSD model, which matches the assumptions of the methods tested, ADMIXTURE is the most accurate followed by SCOPE and ALStructure (Figures 1, S1, S2, S3, and S4). Among the scalable methods, TeraStructure and SCOPE, SCOPE tends to be more accurate in terms of both Jensen-Shannon divergence (JSD) (Table 1) and root-mean-square error (RMSE) (Table 2). We also assessed accuracy under a spatial model, which violates the assumptions of the PSD model by inducing a spatial relationship between the admixture proportions (Figures 2, S5, S6, and S7). Under this scenario, SCOPE, ALStructure, and sNMF are typically the most accurate (Tables 1 and 2).

**Table 1:** Jensen-Shannon divergence measurements for methods on simulated data

**Dataset type**	**Base dataset**	**k**	**n**	**m**	**ADMIXTURE**	**fastStructure**	**TeraStructure**	**ALStructure**	**sNMF**	**SCOPE**
PSD	HGDP	6	10,000	10,000	2.4^∗^	6.3	13.7	3.6	2.4^∗^	3.6
PSD	TGP	6	10,000	10,000	0.8^∗^	11.3	8.8	1.9	2.4	1.9
PSD	TGP	6	10,000	1,000,000	0.03^∗^	8.1	0.2	–	–	0.2
PSD	TGP	6	100,000	1,000,000	–	–	0.3	–	–	0.2^∗^
PSD	TGP	6	1,000,000	1,000,000	–	–	–	–	–	0.2^∗^
Spatial	HGDP	6	10,000	10,000	6.5	33.9	5.7	2.1^∗^	2.3	2.6
Spatial	TGP	6	10,000	10,000	6.8	31.1	3.4	2.4^∗^	4.0	3.3
Spatial	TGP	10	10,000	100,000	12.4	34.7	6.3	8.1	5.7	5.6^∗^
Spatial	TGP	10	10,000	1,000,000	–	–	10.0	–	–	8.2^∗^

Jensen-Shannon divergence (JSD) was computed against the ground truth admixture proportions for each simulation. Values are displayed as percentages rounded to one decimal place. Estimated proportions of 0 were set to $1\times 10^{-9}$ (see subjects and methods). A dash denotes that the method was not run because of projected time or memory usage. Values with an asterisk denote the best value for each dataset.

**Table 2:** Root-mean-square error measurements for methods on simulated data

**Dataset type**	**Base dataset**	**k**	**n**	**m**	**ADMIXTURE**	**fastStructure**	**TeraStructure**	**ALStructure**	**sNMF**	**SCOPE**
PSD	HGDP	6	10,000	10,000	4.0^∗^	10.3	16.6	5.6	4.1	5.6
PSD	TGP	6	10,000	10,000	1.8^∗^	15.9	13.7	3.2	4.1	3.2
PSD	TGP	6	10,000	1,000,000	0.2^∗^	12.4	0.9	–	–	0.3
PSD	TGP	6	100,000	1,000,000	–	–	1.0	–	–	0.4^∗^
PSD	TGP	6	1,000,000	1,000,000	–	–	–	–	–	0.5^∗^
Spatial	HGDP	6	10,000	10,000	11.9	31.1	10.2	5.7^∗^	5.7^∗^	6.5
Spatial	TGP	6	10,000	10,000	12.5	29.1	6.8^∗^	7.5	9.4	7.3
Spatial	TGP	10	10,000	100,000	10.8	22.8	8.8	8.5	6.7^∗^	6.7^∗^
Spatial	TGP	10	10,000	1,000,000	–	–	6.6^∗^	–	–	7.2

Root-mean-square error (RMSE) was computed against the ground truth admixture proportions for each simulation. RMSE is displayed in percentage and rounded to the first decimal place. A dash denotes that the method was not run due to projected time or memory usage. Values with an asterisk denote the best value for each dataset.

We also observe similar trends when calculating Kullback-Leibler (KL) divergence (Tables S1 and S2) but opt to use JSD as a primary accuracy measurement because of the asymmetric nature of KL divergence, which changes depending on the order of inputs. We also assessed whether SCOPE can consistently arrive at similar solutions across runs regardless of the stochastic approximations used in SCOPE’s algorithm. We ran five replicates of SCOPE from 2–40 inferred populations on a HGDP PSD simulation (Figure S8A), TGP PSD simulation (Figure S8B), HGDP dataset (Figure S8C), and HO dataset (Figure S8D). We observe in our simulated datasets that SCOPE is consistent across both JSD and RMSE between solutions up to the simulated number of populations. Both accuracy measures decrease when inferred more populations than simulated. For the HGDP and HO datasets, we observed that SCOPE is mostly consistent even up to 40 inferred populations. On occasion, we see slight inconsistency, but this is largely because one replicate differed from the other (Figure S9).

### Runtime and memory

Using simulated and real datasets, we compared the runtime of SCOPE to ADMIXTURE, fastStructure, TeraStructure, sNMF, and ALStructure (Table 3). Not all of the compared methods could be run on all datasets within practical constraints of time and memory. On the largest PSD datasets that each method could be run on, SCOPE is over 150 times faster than ADMIXTURE (10,000 individuals by 1 million SNPs), over 500 times faster than fastStructure (10,000 individuals by 1 million SNPs), about 100 times faster than ALStructure (10,000 individuals by 100,000 SNPs), over 110 times faster than TeraStructure (100,000 individuals by 1 million SNPs), and as fast as sNMF (10,000 individuals by 10,000 SNPs). SCOPE is also capable of running on a dataset containing one million SNPs and individuals in just over 24 h (≈ 1 day), whereas TeraStructure is extrapolated to require about 500 h (≈ 20 days) on the basis of times reported in its manuscript^7^ as well as our experiments (see “benchmarking” sections of subjects and methods).

**Table 3:** Runtimes and fold-speedups of methods on simulations and real datasets

**Dataset type**	**Base dataset**	**k**	**n**	**m**	**ADMIXTURE**	**fastStructure**	**TeraStructure**	**ALStructure**	**sNMF**	**SCOPE**
PSD	HGDP	6	10,000	10,000	0:14 (48)	3:44 (746)	0:11 (36)	0:30 (101)	< 1 min^∗^ (1)	< 1 min^∗^
PSD	TGP	6	10,000	10,000	0:17 (206)	1:22 (987)	0:12 (144)	0:23 (271)	< 1 min^∗^ (1)	< 1 min^∗^
PSD	TGP	6	10,000	1,000,000	35:12 (156)	114:51 (509)	20:31 (91)	–	–	0:14^∗^
PSD	TGP	6	100,000	1,000,000	–	–	237:02 (113)	–	–	2:06^∗^
PSD	TGP	6	1,000,000	1,000,000	–	–	–	–	–	24:37^∗^
Spatial	HGDP	6	10,000	10,000	5:52 (440)	4:06 (308)	0:03 (3)	1:39 (124)	< 1 min^∗^ (1)	< 1 min^∗^
Spatial	TGP	6	10,000	10,000	3:11 (239)	3:19 (249)	0:07 (9)	1:55 (144)	∼ 1 min^∗^ (1)	< 1 min^∗^
Spatial	TGP	10	10,000	100,000	284:47 (1,808)	33:03 (210)	4:29 (28)	24:51 (158)	0:33 (4)	0:09^∗^
Spatial	TGP	10	10,000	1,000,000	–	–	15:22 (9)	–	–	1:47^∗^
Real	HGDP	10	940	642,951	4:24 (31)	4:39 (33)	0:40 (5)	0:55 (7)	0:16 (2)	0:08^∗^
Real	HO	14	1,931	385,089	13:28 (122)	24:49 (224)	1:37 (15)	2:11 (20)	0:30 (4)	0:07^∗^
Real	TGP	8	1,718	1,854,622	31:33 (33)	8:53 (9)	4:20 (5)	11:16 (12)	–	0:57^∗^
Real	UKB	4	488,363	569,346	–	–	–	–	–	25:57^∗^
Real	UKB	20	488,363	147,604	–	–	–	–	–	23:42^∗^
Real	UKB	40	488,363	147,604	–	–	–	–	–	51:25^∗^

ADMIXTURE, TeraStructure, sNMF, and SCOPE were run with eight threads. ALStructure and fastStructure were run on a single thread because of their lack of multithreading implementations. Default parameters were used. TeraStructure’s “--rfreq” parameter was set to 10% of the number of SNPs. Times are rounded to the nearest minute and displayed in h:min. The fold-speedup (runtime of method in seconds divided by runtime of SCOPE in seconds) achieved by SCOPE is denoted with each time in parentheses and rounded to the nearest integer. Values with an asterisk denote the best value for each dataset. Runtimes for SCOPE under one minute are denoted as “< 1 min.” A dash denotes that the method was not run because of projected time or memory usage.

The runtime of all methods increases under the spatial model. In this scenario, SCOPE is over 1,800 times faster than ADMIXTURE (10,000 individuals by 100,000 SNPs), about 210 times faster than fastStructure (10,000 individuals by 100,000 SNPs), over 155 times faster than ALStructure (10,000 individuals by 100,000 SNPs), about nine times faster than TeraStructure (10,000 individuals by 1 million SNPs), and four times faster than sNMF (10,000 individuals by 100,000 SNPs) on the largest dataset each method could be run on. Over all of the datasets, SCOPE is up to 1,800 times faster than existing methods and three to 144 times faster than TeraStructure. Furthermore, SCOPE scales linearly with the number of latent populations inferred (Figure S10). Additional threads can also be used by SCOPE to speed up runtime up until a fundamental I/O bound is reached (Figure S11).

SCOPE has a reasonable memory footprint: for large datasets for which only TeraStructure and SCOPE were feasible, SCOPE uses slightly less memory than TeraStructure. The memory usage of SCOPE also scales linearly in the size of genotype matrix (i.e., the number of individuals times the number of SNPs) (Table S3). SCOPE requires less than 250 GB for the UK Biobank dataset (488,363 individuals and 569,346 SNPs) and 750 GB for the dataset consisting of one million individuals and SNPs. When using smaller SNP sets such as the UK Biobank’s PCA set (147,604 SNPs), SCOPE uses about 60 GB of memory (488,363 individuals and 147,604 SNPs).

### Accuracy of supervised analysis

**Figure 3:**
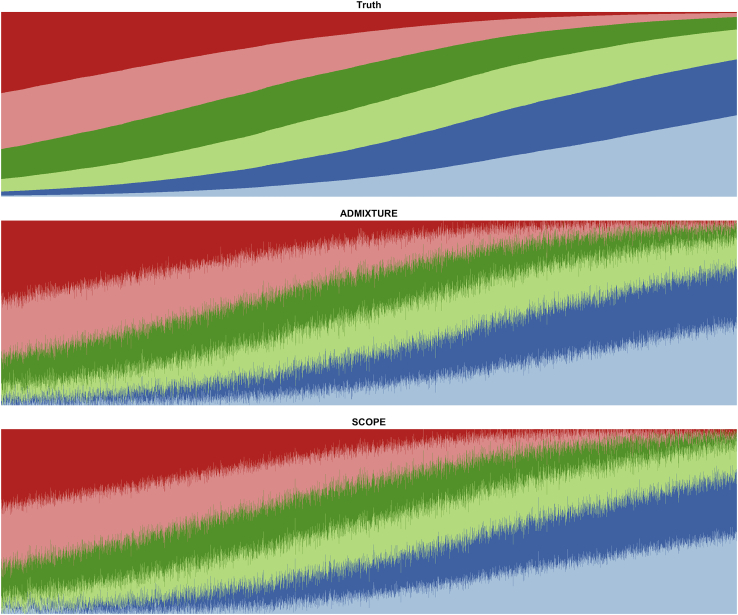
Supervised population structure inference for simulations under a spatial model generated with 1000 Genomes Phase 3 data Model parameters were drawn from TGP data to generate a simulation dataset with 10,000 samples and 10,000 SNPs under a spatial model. Both methods were provided the true population allele frequencies as input. Colors and order of samples are matched between each method to the truth.

Out of the methods tested, only SCOPE and ADMIXTURE are able to use supplied allele frequencies to perform population structure inference in a supervised fashion (Tables 4 and S4). In the PSD model simulations, we observe a small improvement to both RMSE and JSD relative to unsupervised population structure inference (Figures S12, S13, S14, S15, and S16). Under the spatial model simulations, the use of supervision obtains much greater accuracy compared to unsupervised inference (Figures 3, S17, S18, and S19).

**Table 4:** Accuracy of supervised population structure inference with supplied allele frequencies on simulations

**Dataset type**	**Base dataset**	**k**	**n**	**m**	**Supervised**		**Unsupervised**	
					**RMSE**	**JSD**	**RMSE**	**JSD**
PSD	HGDP	6	10,000	10,000	2.9^∗^	1.5^∗^	5.6	3.6
PSD	TGP	6	10,000	10,000	2.0^∗^	0.9^∗^	3.2	1.9
PSD	TGP	6	10,000	1,000,000	0.2^∗^	0.1^∗^	0.3	0.2
PSD	TGP	6	100,000	1,000,000	0.2^∗^	0.1^∗^	0.4	0.2
PSD	TGP	6	1,000,000	1,000,000	0.2^∗^	0.1^∗^	0.5	0.2
Spatial	HGDP	6	10,000	10,000	2.4^∗^	0.6^∗^	6.5	2.6
Spatial	TGP	6	10,000	10,000	1.7^∗^	0.3^∗^	7.3	3.3
Spatial	TGP	10	10,000	100,000	0.6^∗^	0.3^∗^	6.7	5.6
Spatial	TGP	10	10,000	1,000,000	0.3^∗^	0.1^∗^	8.2	7.2

True allele frequencies were supplied to SCOPE to use in supervised population structure inference. Root-mean-square error (RMSE) and Jensen-Shannon divergence (JSD) were computed against the true admixture proportions. Estimated proportions of 0 were set to $1\times 10^{-9}$ for JSD calculations (see subjects and methods). Values are displayed in percentages and rounded to the first decimal place. Values with an asterisk denote the best value for each dataset.

### Application to real genotype data

**Figure 4:**
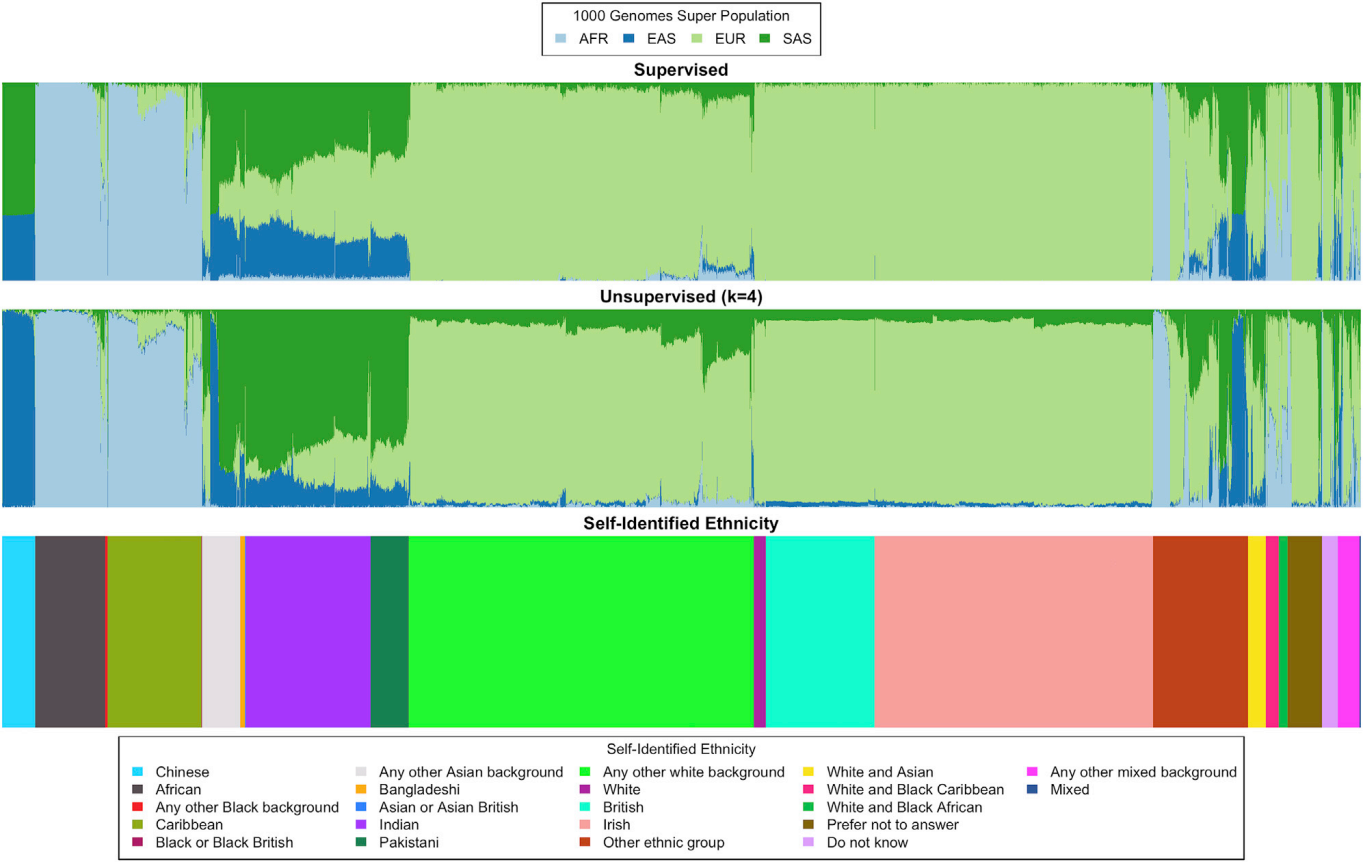
Continental ancestry inference on the UK Biobank We ran population structure inference by using SCOPE on the UK Biobank (488,363 individuals and 569,346 SNPs) both supervised with 1000 Genomes Phase 3 allele frequencies (top) and unsupervised with four latent populations (middle). For reference, we plot the self-identified race/ethnicity (bottom). For visualization purposes, we reduced the number of self-identified British individuals to a random subset of 5,000 individuals. Colors and order of samples are matched between each row of the figure. The full figure without individuals removed can be found in Figure S23.

We applied SCOPE to several real, genomic datasets: TGP (1,718 individuals and 1,184,622 SNPs) with eight latent populations (k=8) (Figure S20), HGDP (940 individuals and 642,951 SNPs) with ten populations (k=10) (Figure S21), Human Origins (HO) (1,931 individuals and 385,089 SNPs)^20^ with 14 populations (k=14) (Figure S22), the UK Biobank (488,363 individuals and 569,346 SNPs) with four populations (k=4) (Figure 4), and the UK Biobank (488,363 individuals and 147,604 SNPs) with 20 populations (k=20) (Figure S24) and 40 populations (k=40) (Figure S25) (see subjects and methods for quality control). We chose the number of latent populations to be consistent with previous studies on these datasets.^7^^,^^8^ For the UK Biobank analysis, we chose four latent populations to infer continental ancestry groups for the larger SNP set and 20 and 40 latent populations to explore SCOPE’s ability to infer larger numbers of latent populations on real data. In terms of runtime and memory, we continued to observe trends consistent with our simulations where SCOPE is orders of magnitude faster than other methods while consuming reasonable amounts of memory (Tables 3 and S3). We note that the runtime for inference on the larger UK Biobank dataset is about the same as the runtime for our 1 million individual and SNP simulation despite the fact that the UK Biobank dataset is approximately a quarter of its size, consistent with the increase in runtimes with model deviations as seen in the context of spatial simulations.

Because there is no ground truth to assess accuracy on these datasets, we used concordance between SIRE and inferred admixture proportions as a metric. We trained multinomial logistic regression models to predict continental ancestry for the TGP (five populations) and HGDP (seven populations) by using the inferred admixture proportions from each method (Table S5). We find that all methods perform similarly on both datasets. For the UK Biobank, SCOPE is able to obtain 88.27% accuracy when using labels provided by UK Biobank (22 labels) and 95.75% accuracy when ambiguous/heterogeneous labels (e.g., “other,” “mixed”) are removed and population labels are collapsed to continental groupings (eight labels). We did not perform this analysis for the HO dataset because several population labels only contained one sample.

We additionally assessed SCOPE’s ability to infer finer population structure by using the British individuals in the UK Biobank. We trained ordinary least-squares models to predict the self-reported birth location GPS coordinate by using the inferred proportions from the different runs of SCOPE under different numbers of latent populations (four, 20, and 40 latent populations) (Table S6). Increasing the number of latent populations generally improves the prediction accuracy when measured through coefficient of determination $\left(R^{2}\right)$. With four latent populations, the $R^{2}$ is 0.007 and 0.008 for latitude and longitude prediction, respectively. This increases to 0.2–0.3 and approximately 0.15 when increasing the number of latent populations to 20 and 40. We also examined the prediction accuracy in terms of residual distance (difference between predicted and reported location). The 95% quantile for the residual distances decreases from ≈ 334 km to ≈ 290 km when increasing the number of inferred populations from four to 20 or 40.

We also utilized the supervised mode of SCOPE by using known population allele frequencies from TGP superpopulations to infer continental ancestry for all individuals in the UK Biobank. We find that the supervised mode of SCOPE largely agreed with the unsupervised inference (Figures 4 and S23).

## Discussion

We have presented SCOPE, a scalable method for inferring population structure from biobank-scale genomic data. We show that SCOPE remains accurate while being scalable in terms of runtime and memory requirements. SCOPE is also able to perform supervised analyses that leverage allele frequency estimates from previous studies to improve interpretability, runtime, and accuracy.

SCOPE enables new analyses by improving the scalability of admixture proportion inference. The inclusion of more individuals and/or genomic sites allows more rare latent population structure to be discovered in addition to improving estimation of the true latent population frequencies. These are often the cases where scaling to biobank-level data becomes a necessity. Furthermore, many admixture tools are often used as an exploratory analysis being run with different numbers of latent populations (i.e., *k*). Being able to perform several runs quickly becomes important for initial analysis.

The use of SCOPE is not without limitations for real data analysis and interpretation. For instance, although larger non-trivial numbers of latent populations (*k*) such as 20 (Figure S24) and 40 (Figure S25) from the UK Biobank explored in this study increase our ability to dissect finer-scale population structure, they remain very difficult to interpret. Furthermore, when exploring these settings, care must be taken to curate a well-defined SNP set. For example, we see a decrease in prediction accuracy when moving from 20 to 40 latent populations in the UK Biobank. This may be attributed to the fact that the UK Biobank’s PCA SNP set was curated to differentiate continental population structure rather than intracontinental structure. We also observed that SCOPE is consistent when inferring a large number of latent populations as exemplified by our replicate studies on the HGDP (Figure S8C) and HO (Figure S8D) datasets, which suggests there is more fine-scale population structure being detected and opens the question of what these latent populations may correspond to. While the ability to use supervised analysis as we did for the UK Biobank can greatly improve interpretability, supervision with SCOPE largely depends on the accuracy of the reference dataset and frequencies used. Finally, there is still the open question of choosing the appropriate number of latent populations (*k*). Although SCOPE allows one to run several different values for *k*, we do not provide any criteria to choose a specific value of *k*. We defer deeper analysis of these questions for future studies.

The methodology used in SCOPE can also be extended in several ways. Several methods that perform structure inference on other genomic datasets^28^^,^^29^ utilize semi-supervised approaches where there are both known and unknown populations. A possible approach for semi-supervision with SCOPE is to perform a multi-stage inference procedure where supervised inference is first applied and unsupervised inference is applied on the residual or unexplained structure. Most current methods, including SCOPE, ignore additional information within the data, such as correlation patterns (i.e., linkage disequilibrium [LD]). Some methods such as fineSTRUCTURE^30^ can perform LD-aware population structure inference but are challenging to scale. The development of methods that can model LD while retaining scalability is a key step in advancing population structure inference.

Though not directly related to the admixture model, there are several approaches to finding broader forms of structure that are not explicitly in the form of admixture proportions. For instance, possible usage of non-linear dimensionality reduction techniques such as UMAP^31^ could provide promising ways to extend beyond current methods, which solely utilize linear methods such as PCA. Other approaches to detecting fine-scale structure include using identity-by-descent (IBD)^32^ or tree-based methods.^33^ Finding ways to scalably bridge these different approaches with the admixture model is still an open question. Finally, extensions of the techniques used in SCOPE can be used to infer relevant structure in other domains such as metagenomics and single-cell transcriptomics.

## Data and code availability

SCOPE can be found at https://github.com/sriramlab/SCOPE. Scripts for simulations, visualization, assessment, downloading of publicly available data, and real data filtering and additional code used in this study can be found at the repository as well. UK Biobank dataset is the only dataset used in this study that is not publicly available but can be obtained by application (https://www.ukbiobank.ac.uk/).

## Web resources

C++ Spectra library, https://spectralib.org/
*lpSolve*, https://CRAN.R-project.org/package=lpSolve

## Supplemental information

Document S1. Figures S1–S25 and Tables S1–S6

Document S2. Document plus supplemental information
