# Supplementary material for: Inferring population structure in biobank-scale genomic data
Source: Am J Hum Genet. 2022 Mar 16;109(4):727–37. doi: 10.1016/j.ajhg.2022.02.015 (PMC9069078; doi:10.1016/j.ajhg.2022.02.015)
Supplement: Document S1. Figures S1–S25 and Tables S1–S6 [file mmc1.pdf]

The American Journal of Human Genetics, Volume *109*

Supplemental information

Inferring population structure in biobank-scale genomic data

Alec M. Chiu, Erin K. Molloy, Zilong Tan, Ameet Talwalkar, and Sriram Sankararaman

Supplemental Information

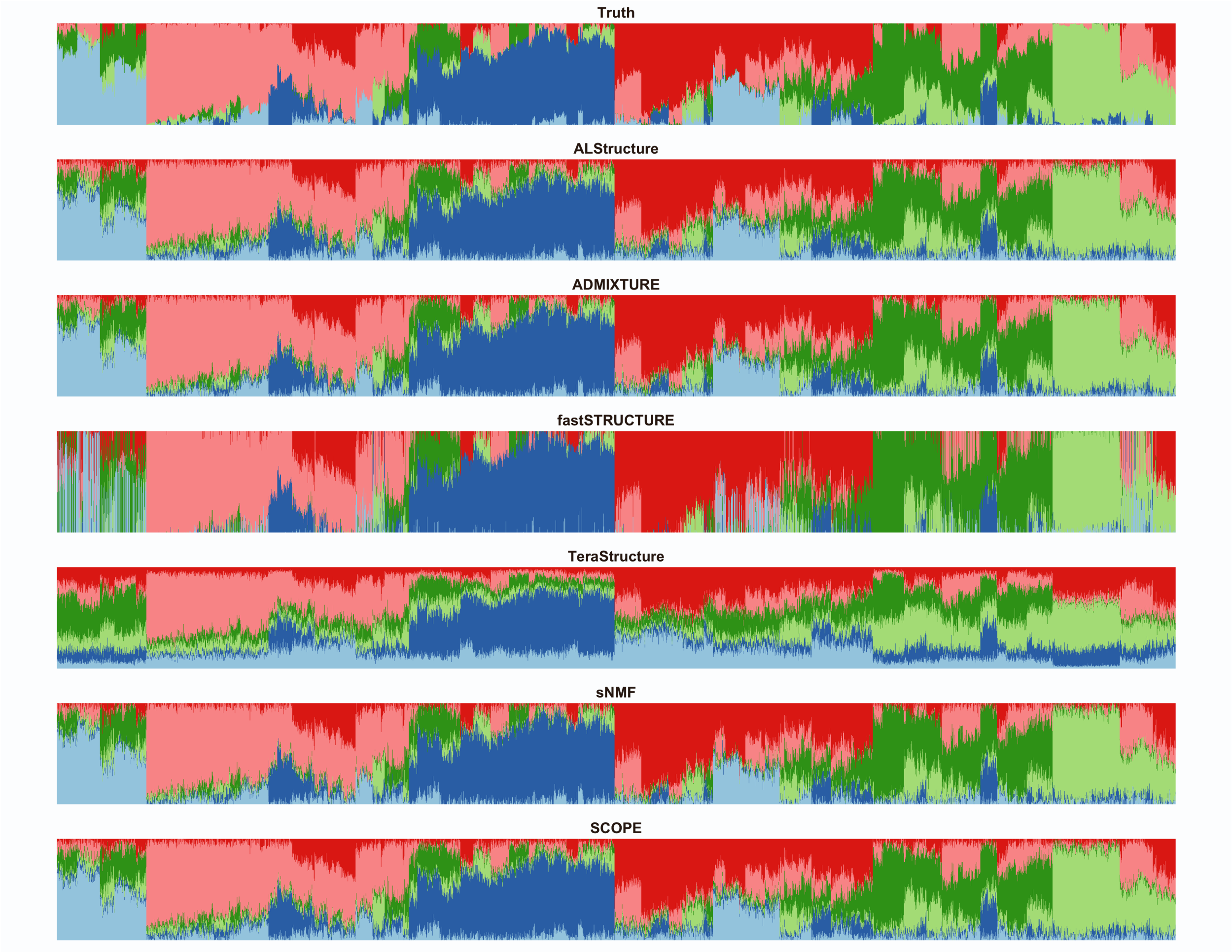

Figure S1: **Population structure inference for simulations under PSD model generated using Human Genomes Diversity Project data.** PSD model parameters were drawn from HGDP data to generate a simulation dataset with 10,000 samples and 10,000 SNPs. The true admixture proportions and resulting inferred admixture proportions from each method are shown. Colors and order of samples are matched between each method to the truth.

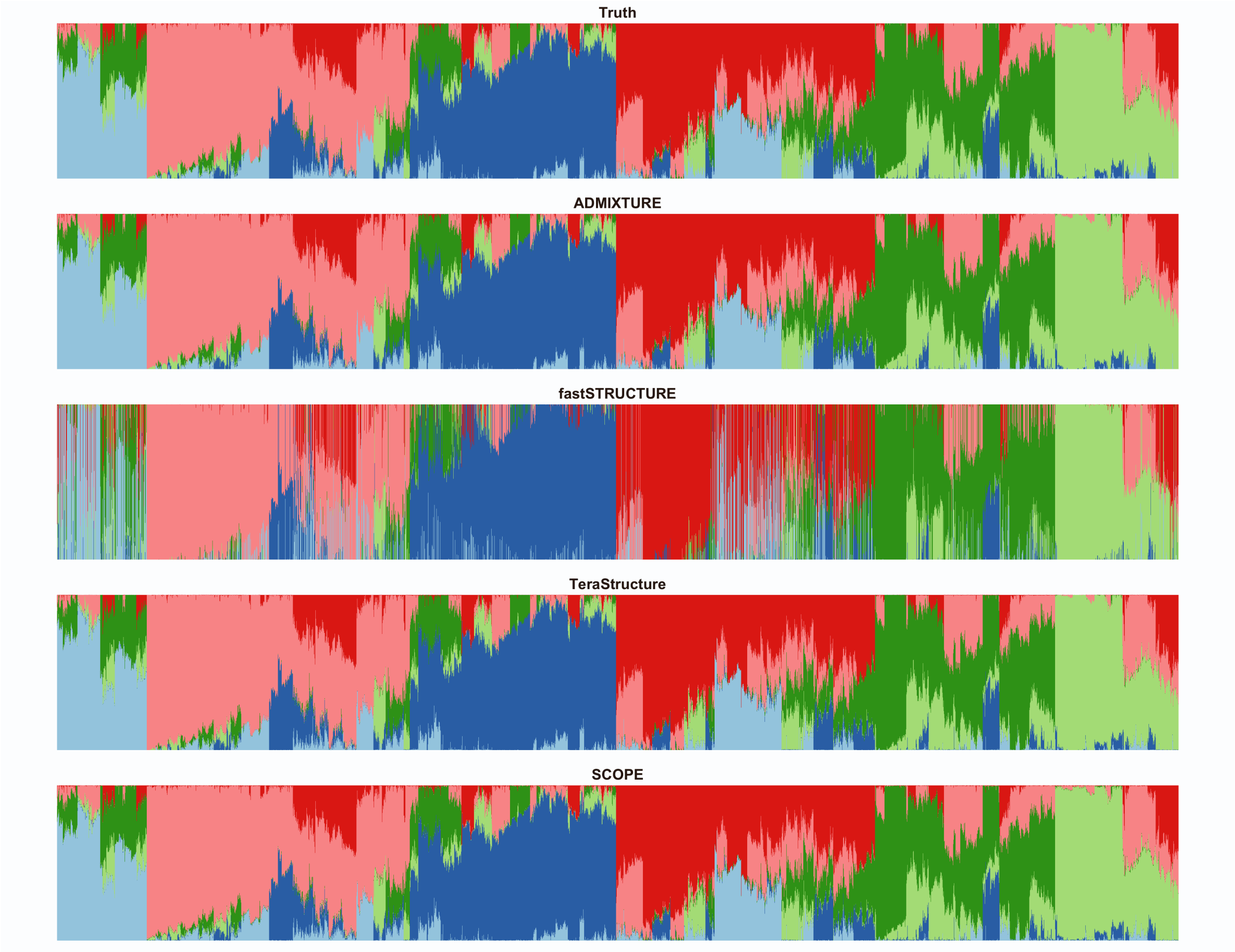

Figure S2: **Population structure inference for simulations under PSD model generated using 1000 Genomes Phase 3 data.** PSD model parameters were drawn from TGP data to generate a simulation dataset with 10,000 samples and 1 million SNPs. The true admixture proportions and resulting inferred admixture proportions from each method are shown. Colors and order of samples are matched between each method to the truth.

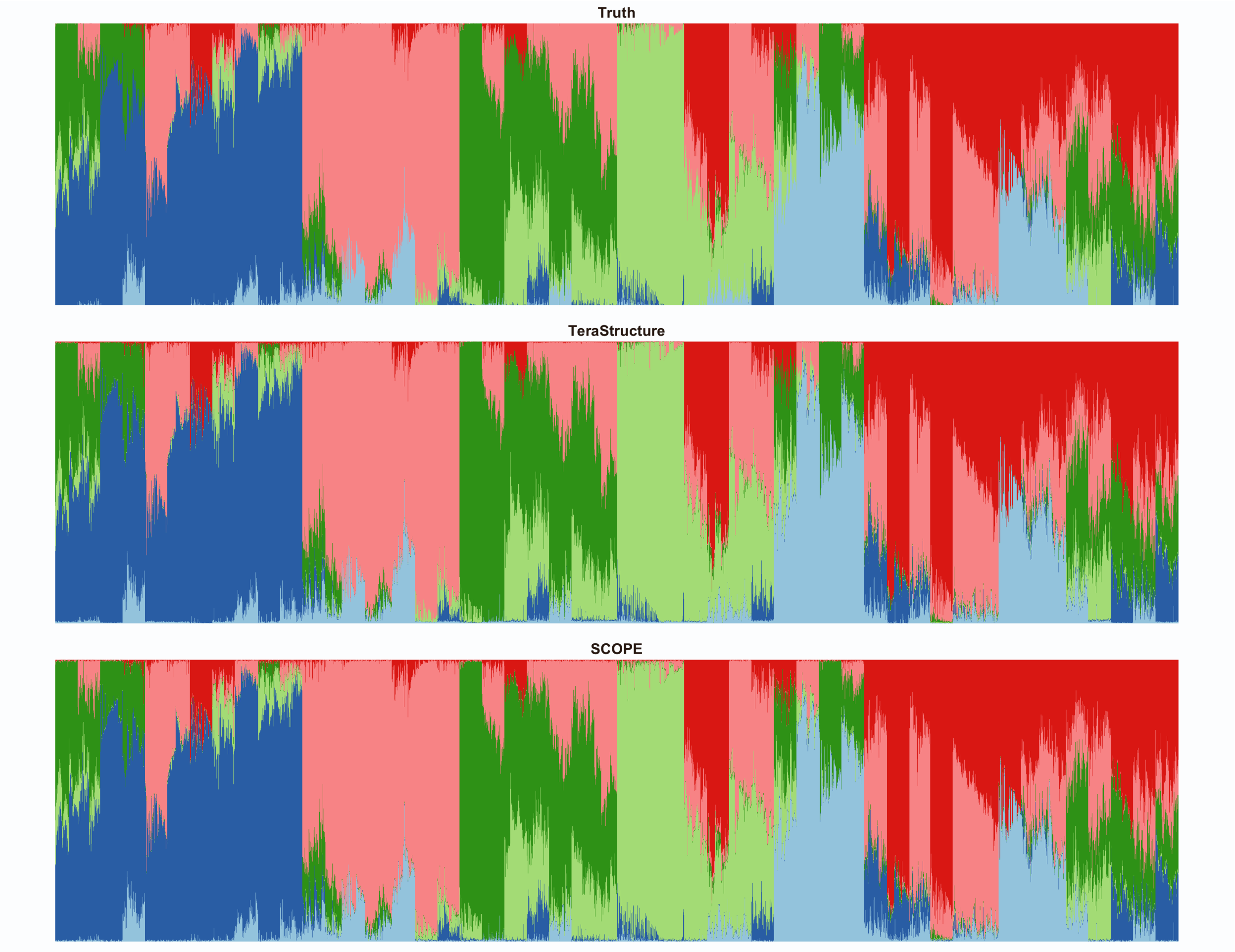

Figure S3: **Population structure inference for simulations under PSD model generated using 1000 Genomes Phase 3 data.** PSD model parameters were drawn from TGP data to generate a simulation dataset with 100,000 samples and 1 million SNPs. The true admixture proportions and resulting inferred admixture proportions from each method are shown. Colors and order of samples are matched between each method to the truth.

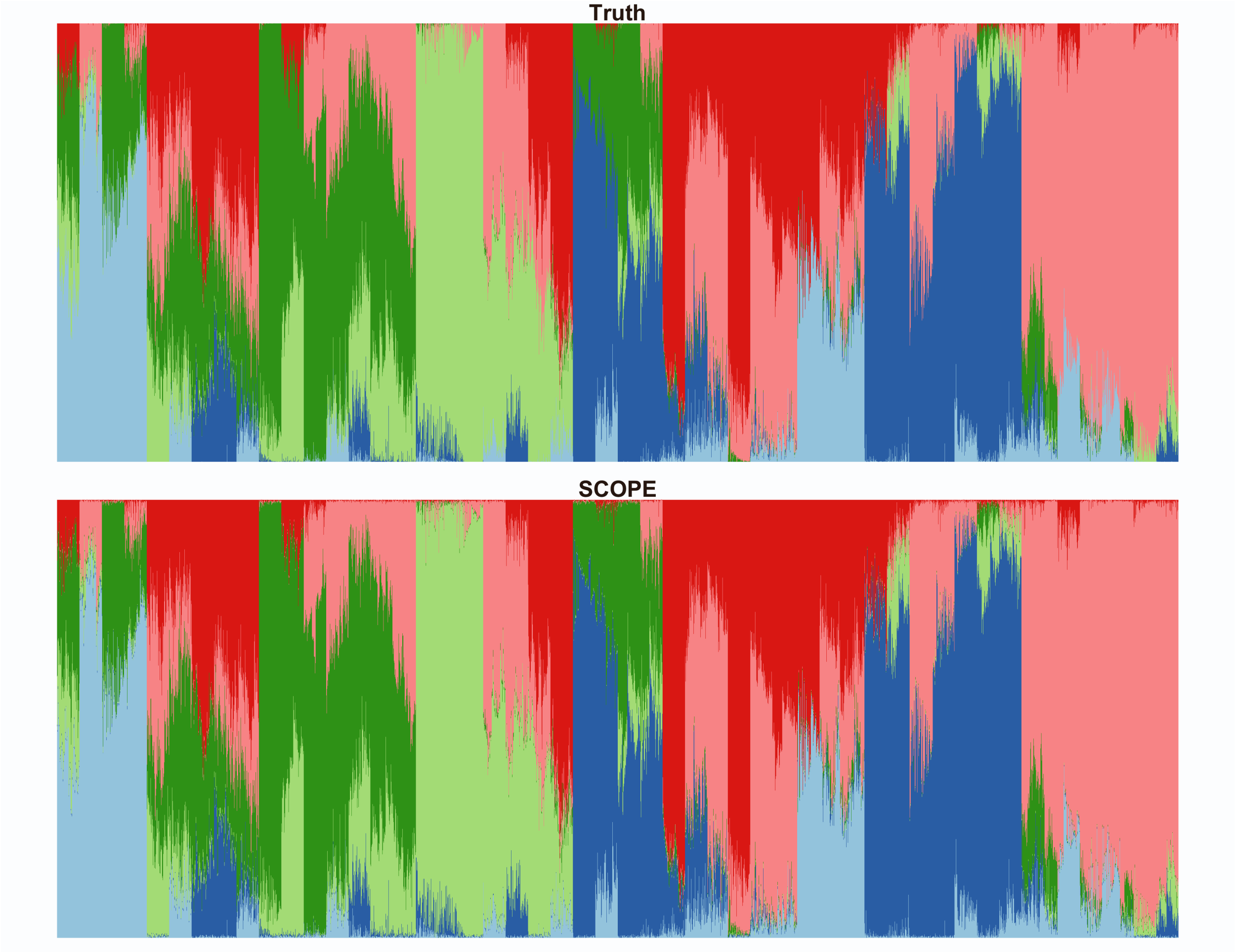

Figure S4: **Population structure inference for simulations under PSD model generated using 1000 Genomes Phase 3 data.** PSD model parameters were drawn from TGP data to generate a simulation dataset with 1 million samples and SNPs. The true admixture proportions and resulting inferred admixture proportions are shown. Colors and order of samples are matched between SCOPE and the true admixture proportions.

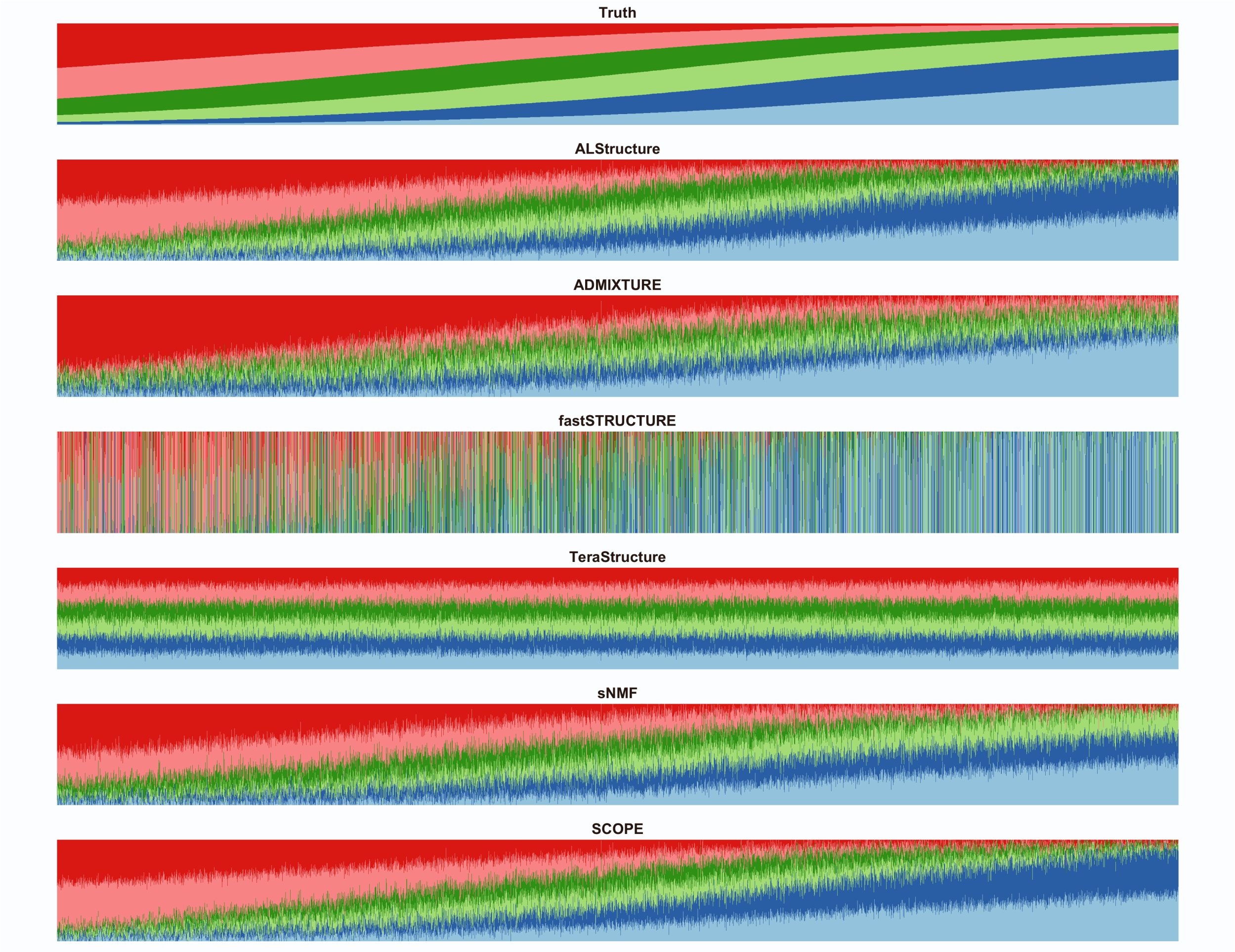

Figure S5: **Population structure inference for simulations under a spatial model generated using Human Genome Diversity Project data.** Model parameters were drawn from HGDP data to generate a simulation dataset with 10,000 samples and 10,000 SNPs under a spatial model (see Methods). The true admixture proportions and resulting inferred admixture proportions from each method are shown. Colors and order of samples are matched between each method to the truth.

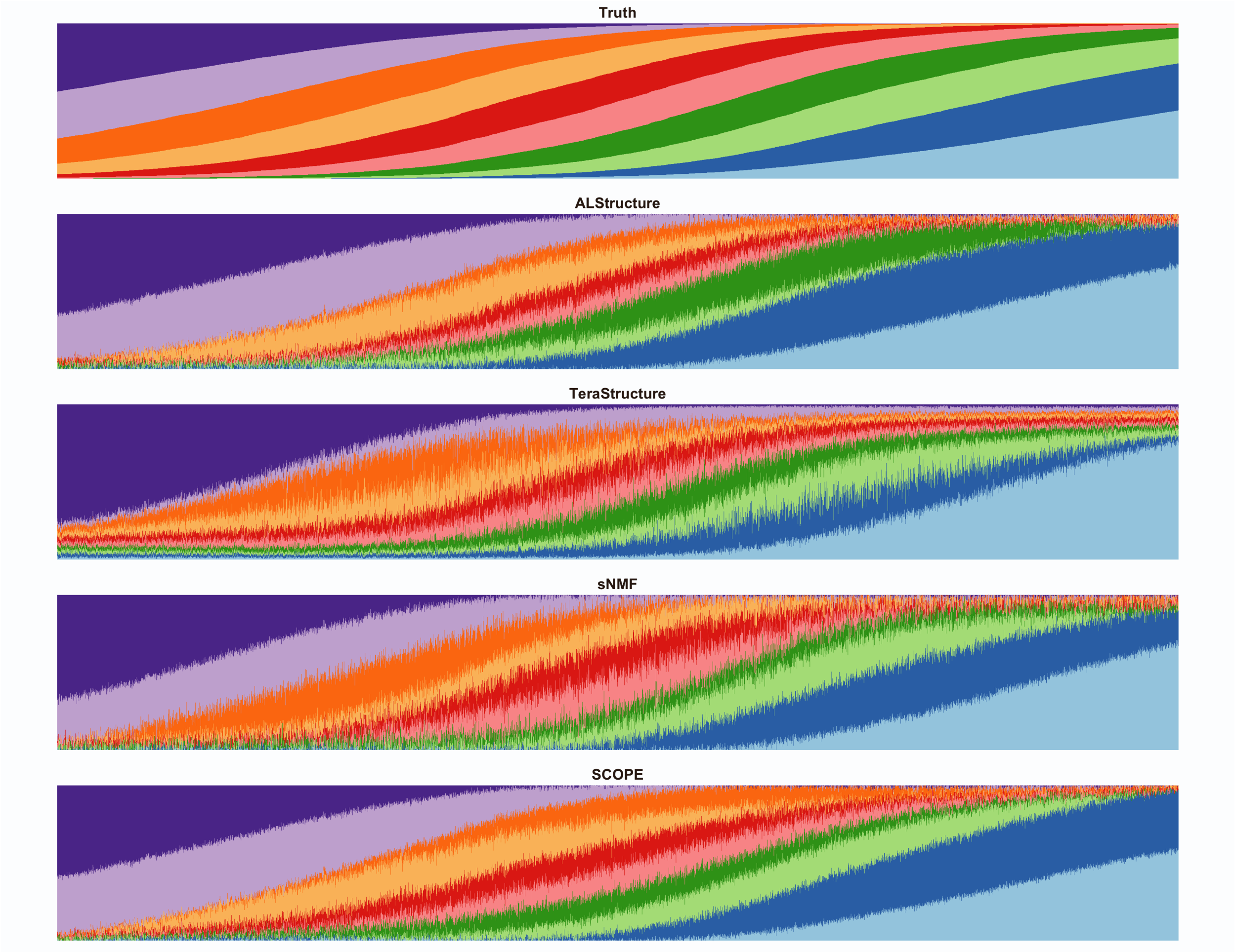

Figure S6: **Population structure inference for simulations under a spatial model generated using 1000 Genomes Phase 3 data.** Model parameters were drawn from TGP data to generate a simulation dataset with 10,000 samples and 100,000 SNPs under a spatial model (see Methods). The true admixture proportions and resulting inferred admixture proportions from each method are shown. Colors and order of samples are matched between each method to the truth.

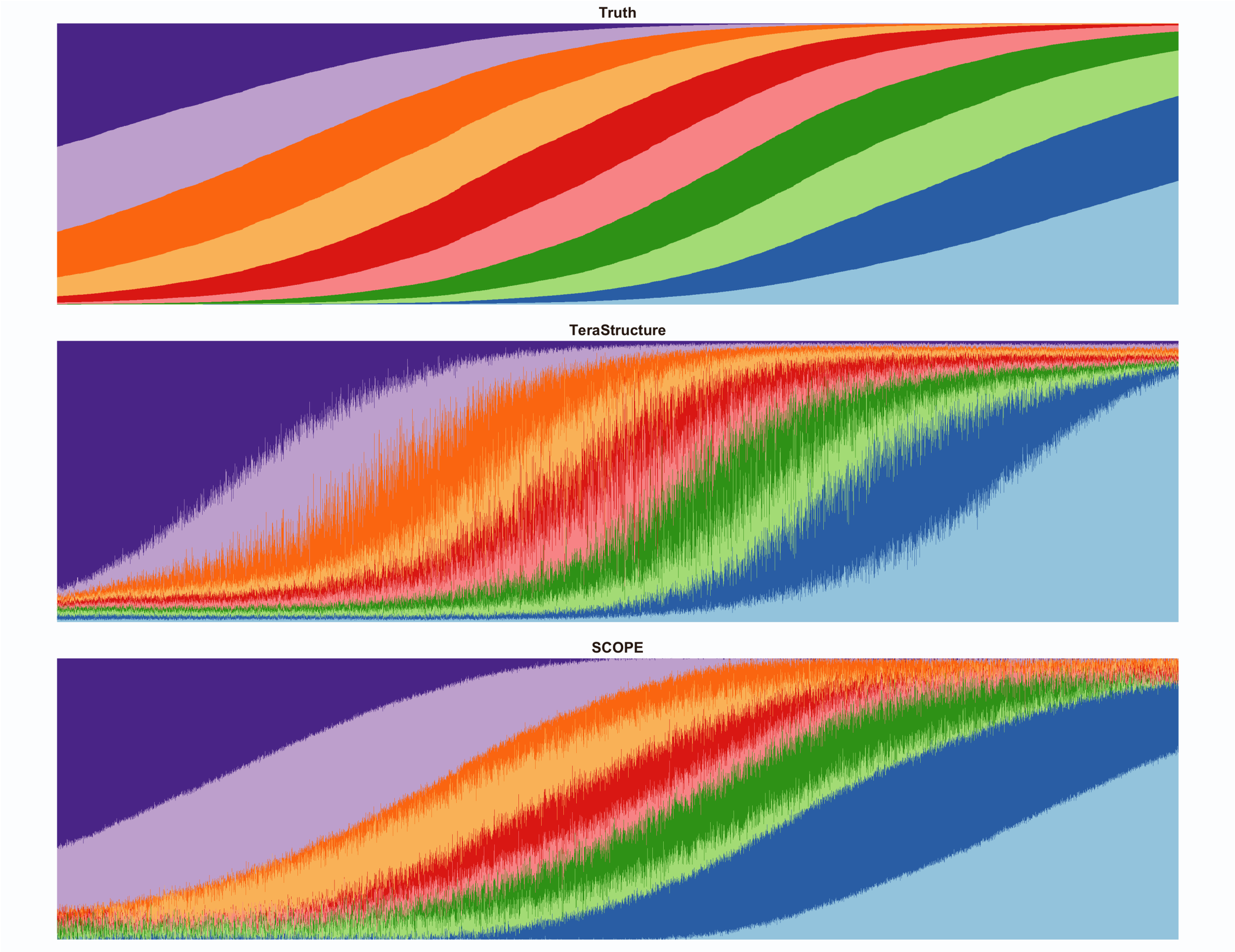

Figure S7: **Population structure inference for simulations under a spatial model generated using 1000 Genomes Phase 3 data.** Model parameters were drawn from TGP data to generate a simulation dataset with 10,000 samples and 1 millions SNPs under a spatial model (see Methods). The true admixture proportions and resulting inferred admixture proportions from each method are shown. Colors and order of samples are matched between each method to the truth.

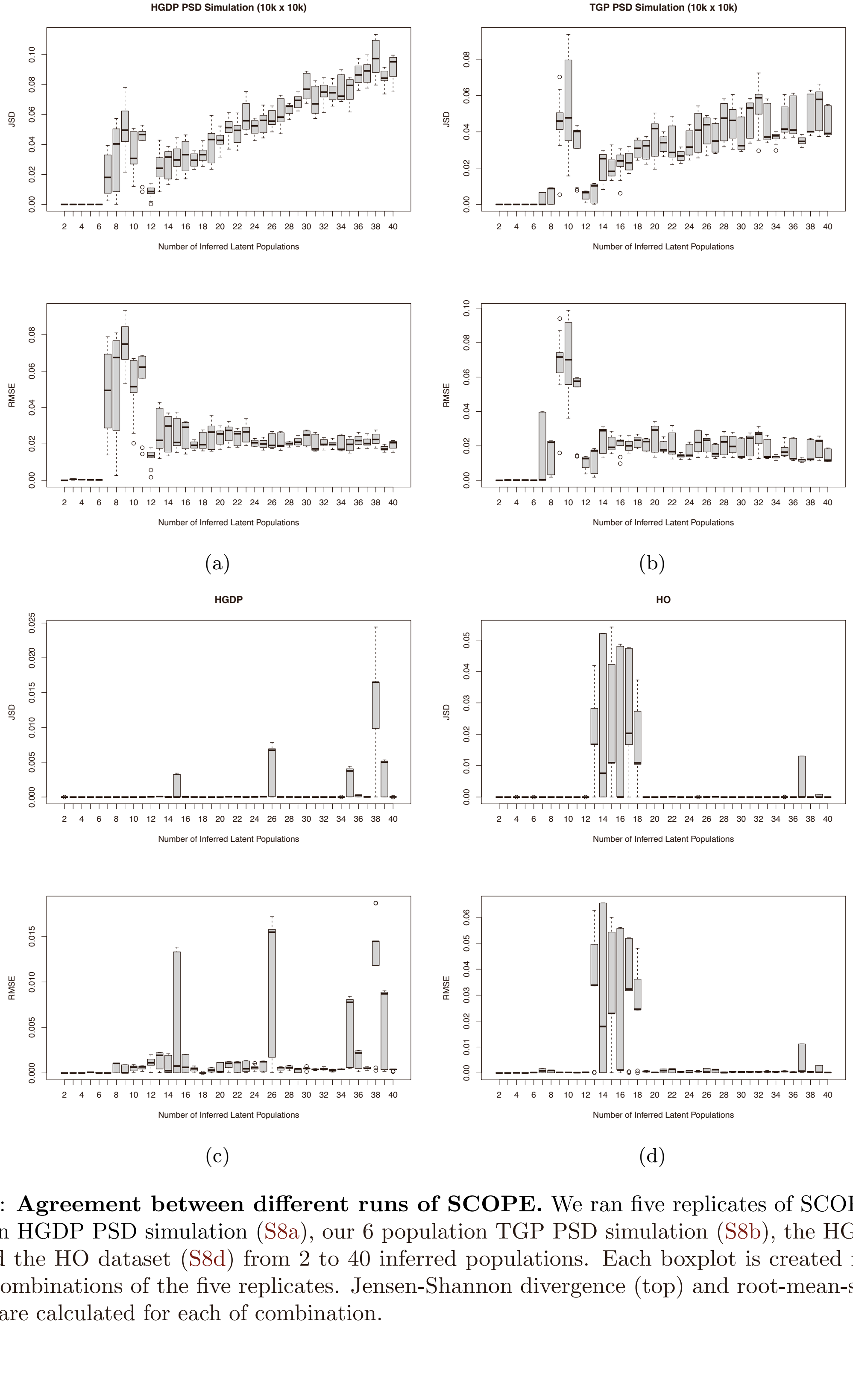

Figure S8: **Agreement between different runs of SCOPE.** We ran five replicates of SCOPE on our 6 population HGDP PSD simulation (S8a), our 6 population TGP PSD simulation (S8b), the HGDP dataset (S8c), and the HO dataset (S8d) from 2 to 40 inferred populations. Each boxplot is created from the 10 possible combinations of the five replicates. Jensen-Shannon divergence (top) and root-mean-square error (bottom) are calculated for each of combination.

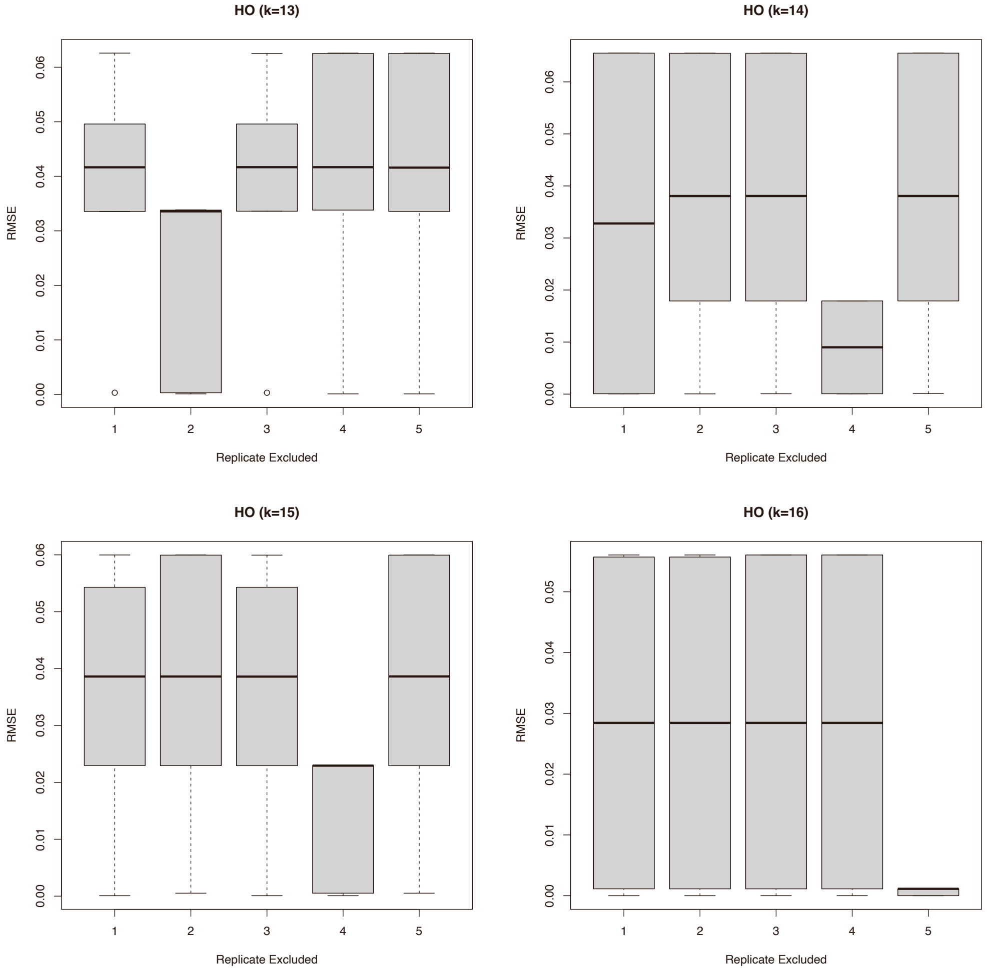

Figure S9: **Excluding one replicate decreases variability between runs.** We repeated the calculations as in Figure S8, but excluded one replicate. When excluding one of the five replicates, the variability between different runs of SCOPE decreases.

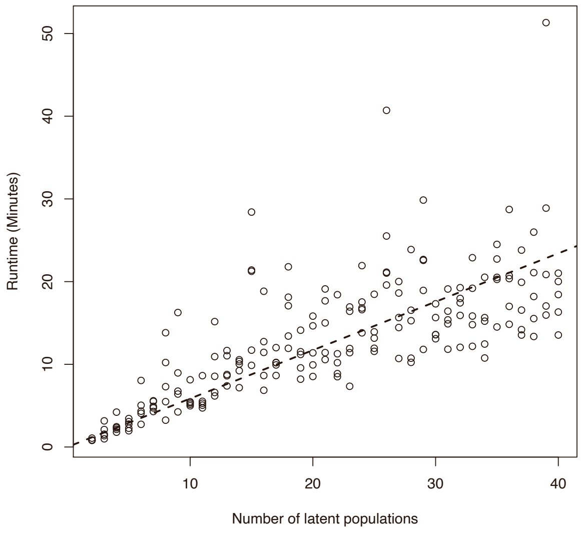

(a)

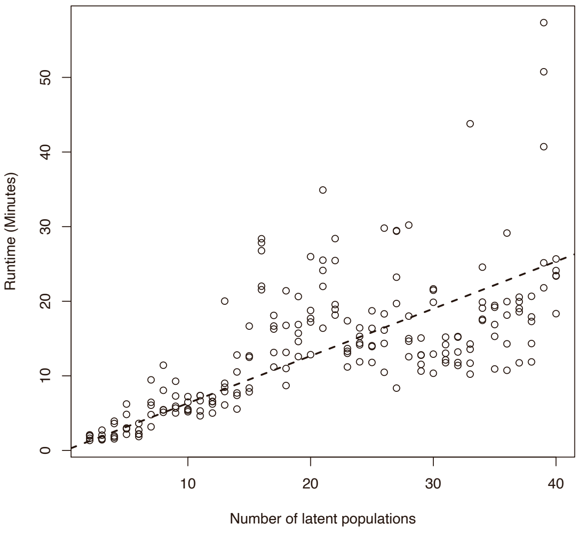

(b)

Figure S10: **Runtime scales linearly with increasing number of latent populations.** SCOPE was run on the HGDP (S10a) and HO (S10b) datasets with 2 to 40 latent populations (k). We ran five replicates for each value of k. The dashed line represents the least squares estimate for each dataset. Each run of SCOPE was performed using 8 threads.

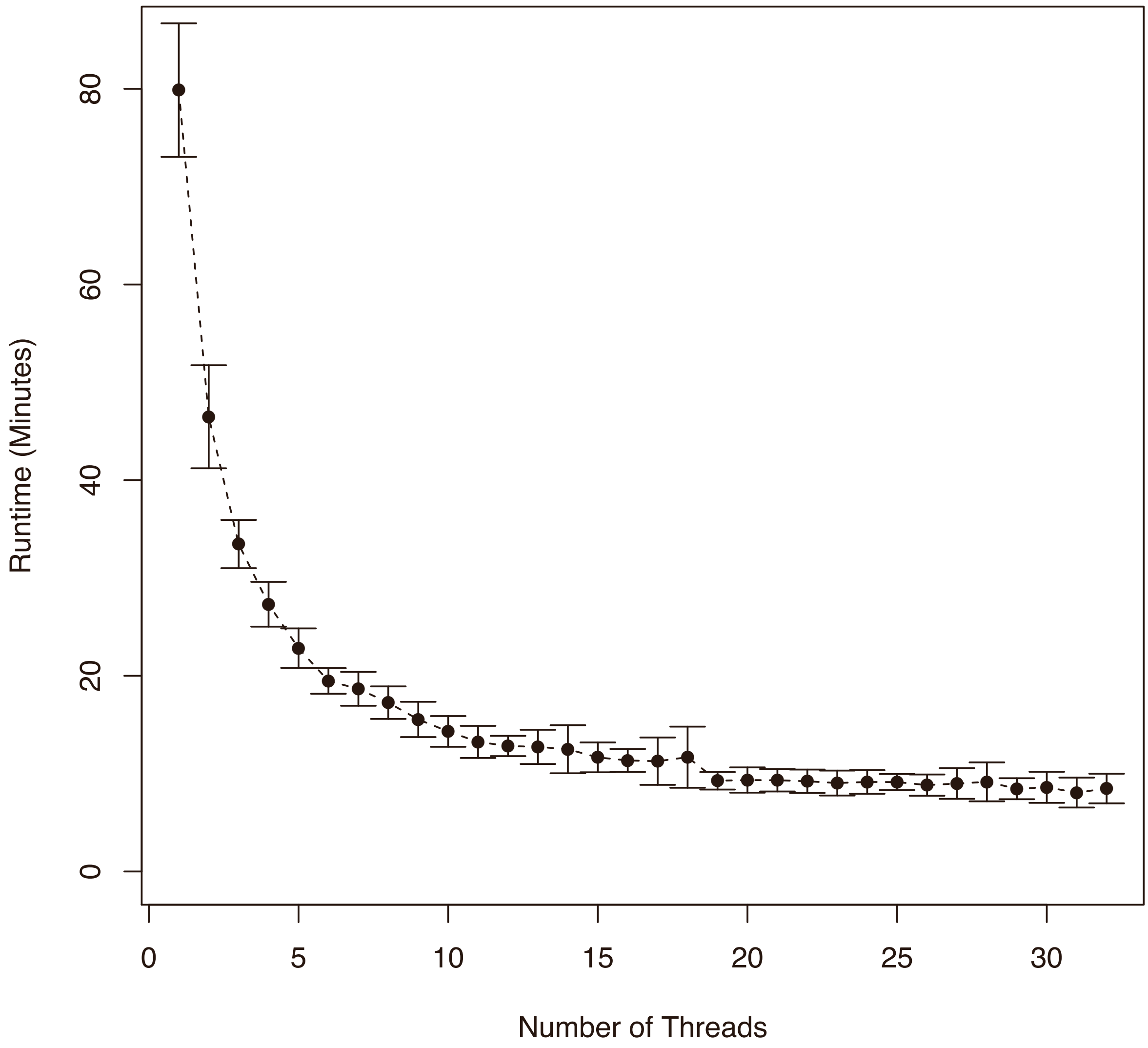

Figure S11: **Runtime scales sublinearly with number of threads.** SCOPE was run on our PSD simulation dataset with 10,000 individuals, 1 million SNPs, and 6 latent populations. We varied the number of threads used from 1-32 and repeated the experiment 5 times for each number of threads. Means and one standard deviation are shown in the figure.

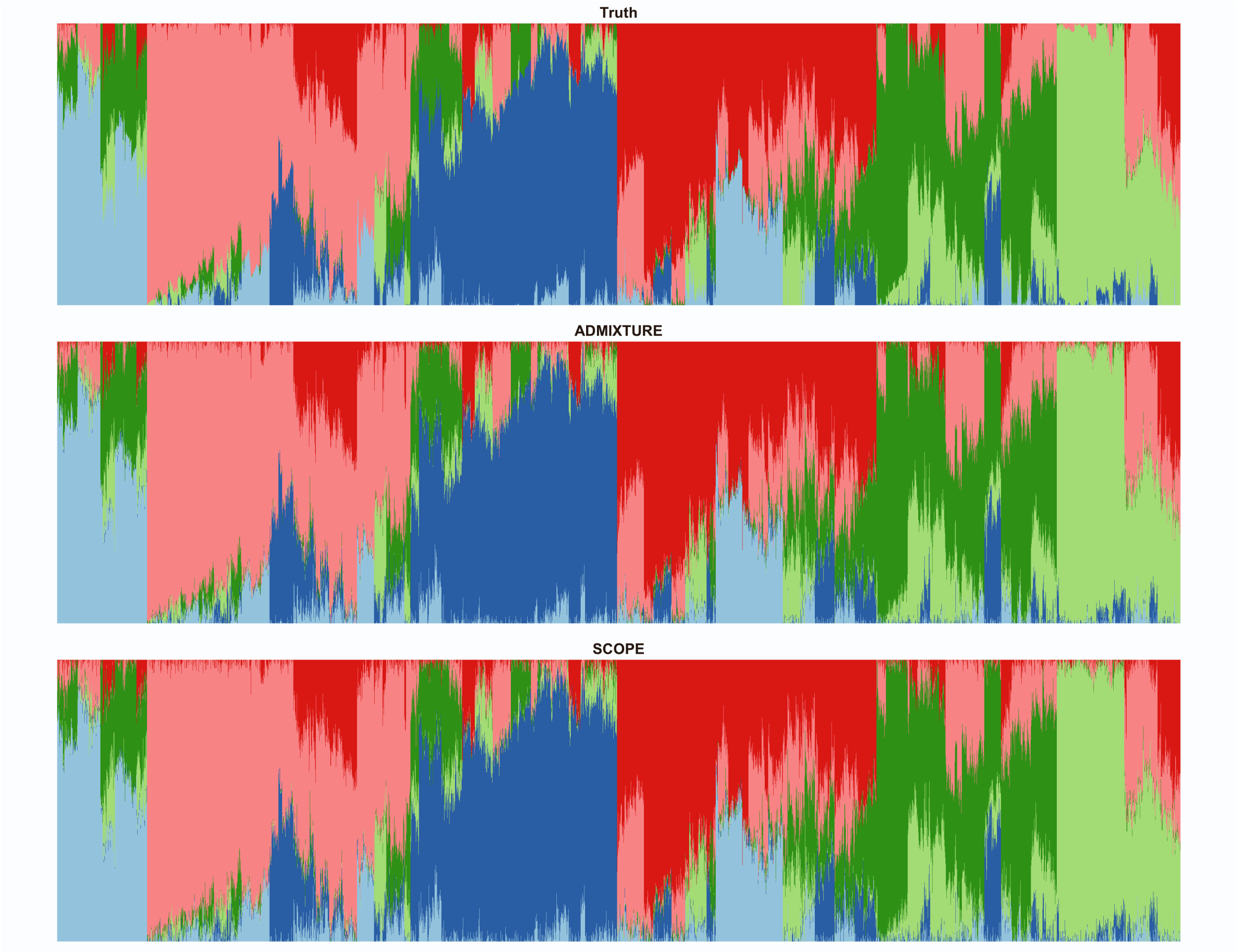

Figure S12: **Supervised population structure inference for simulations under the PSD model generated using 1000 Genomes Phase 3 data.** PSD model parameters were drawn from TGP data to generate a simulation dataset with 10,000 samples and 10,000 SNPs. Both were methods provided the true population allele frequencies as input. Colors and order of samples are matched between each method to the truth.

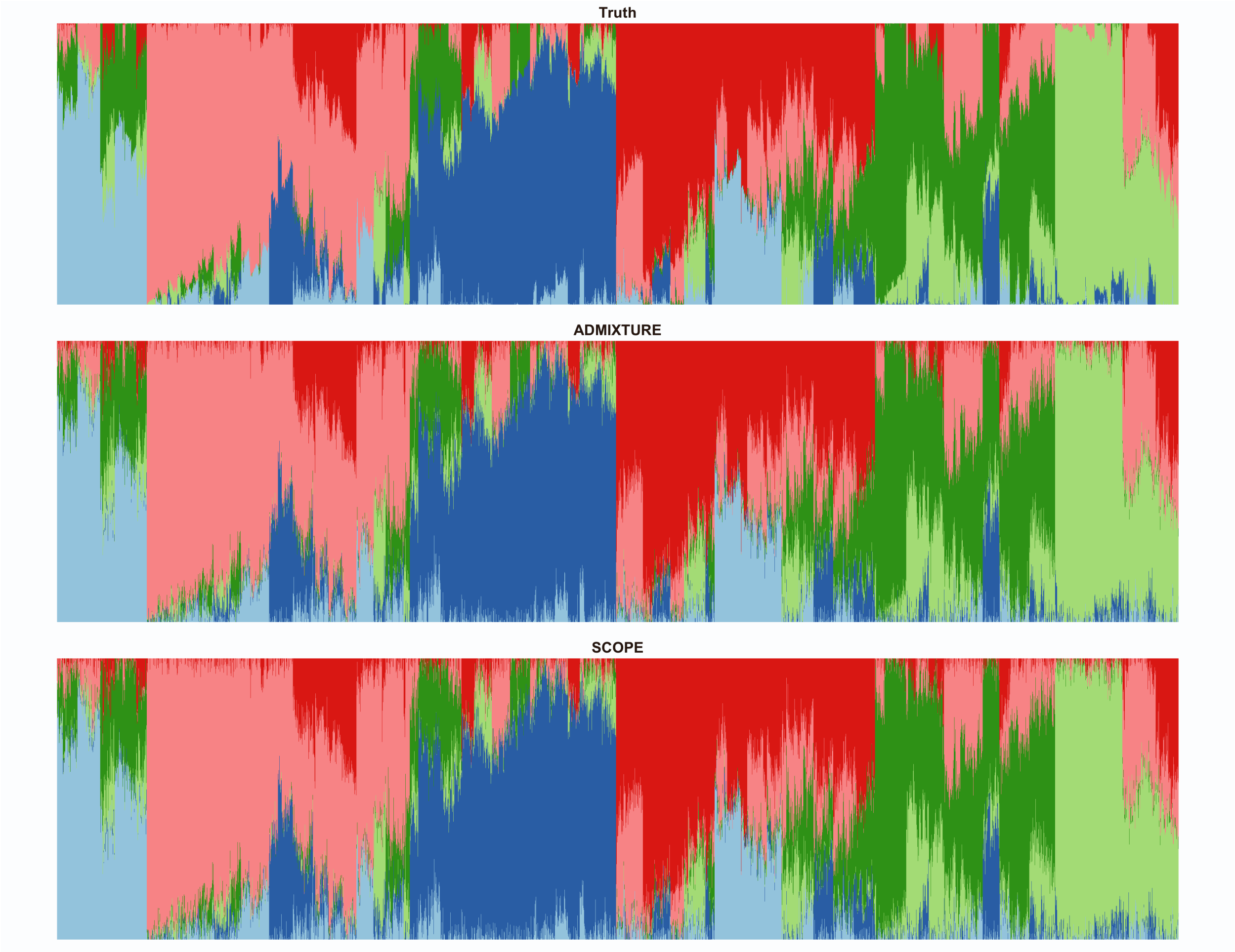

Figure S13: **Supervised population structure inference for simulations under the PSD model generated using Human Genome Diversity data.** PSD model parameters were drawn from HGDP data to generate a simulation dataset with 10,000 samples and 10,000 SNPs. Both were methods provided the true population allele frequencies as input. Colors and order of samples are matched between each method to the truth.

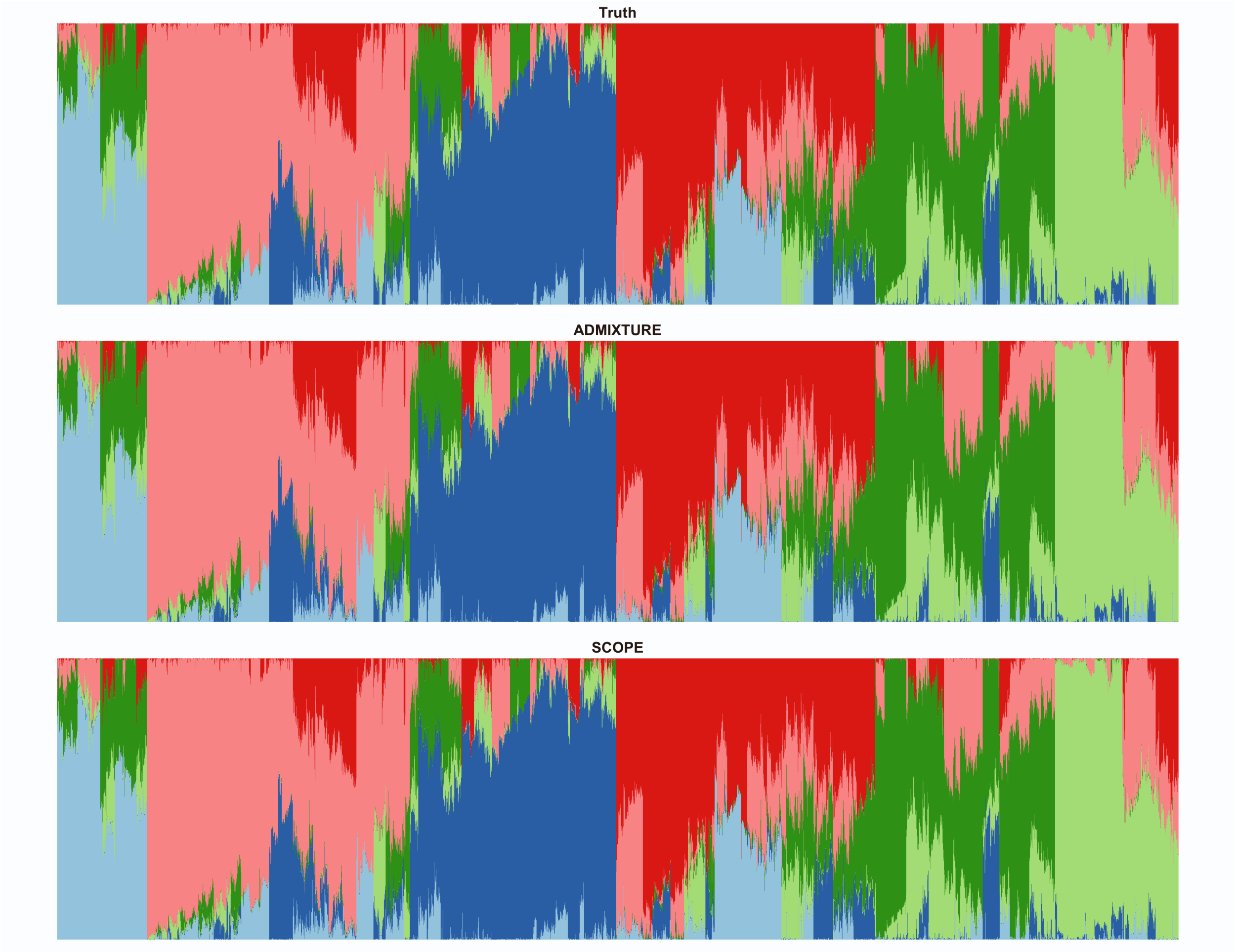

Figure S14: **Supervised population structure inference for simulations under the PSD model generated using 1000 Genomes Phase 3 data.** PSD model parameters were drawn from TGP data to generate a simulation dataset with 10,000 samples and 1 million SNPs. Both were methods provided the true population allele frequencies as input. Colors and order of samples are matched between each method to the truth.

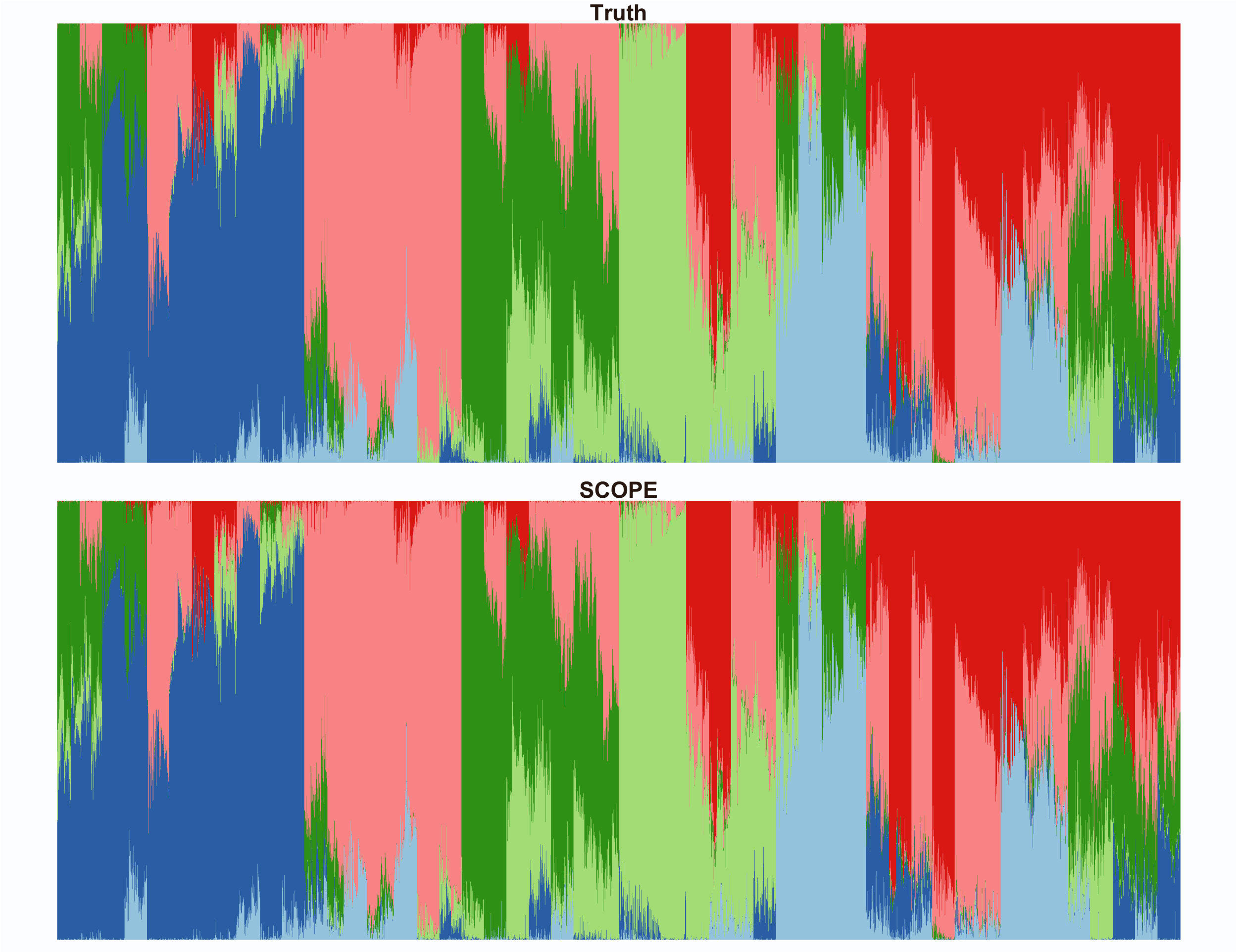

Figure S15: **Supervised population structure inference for simulations under the PSD model generated using 1000 Genomes Phase 3 data.** PSD model parameters were drawn from TGP data to generate a simulation dataset with 100,000 samples and 1 million SNPs. Both were methods provided the true population allele frequencies as input. Colors and order of samples are matched between each method to the truth.

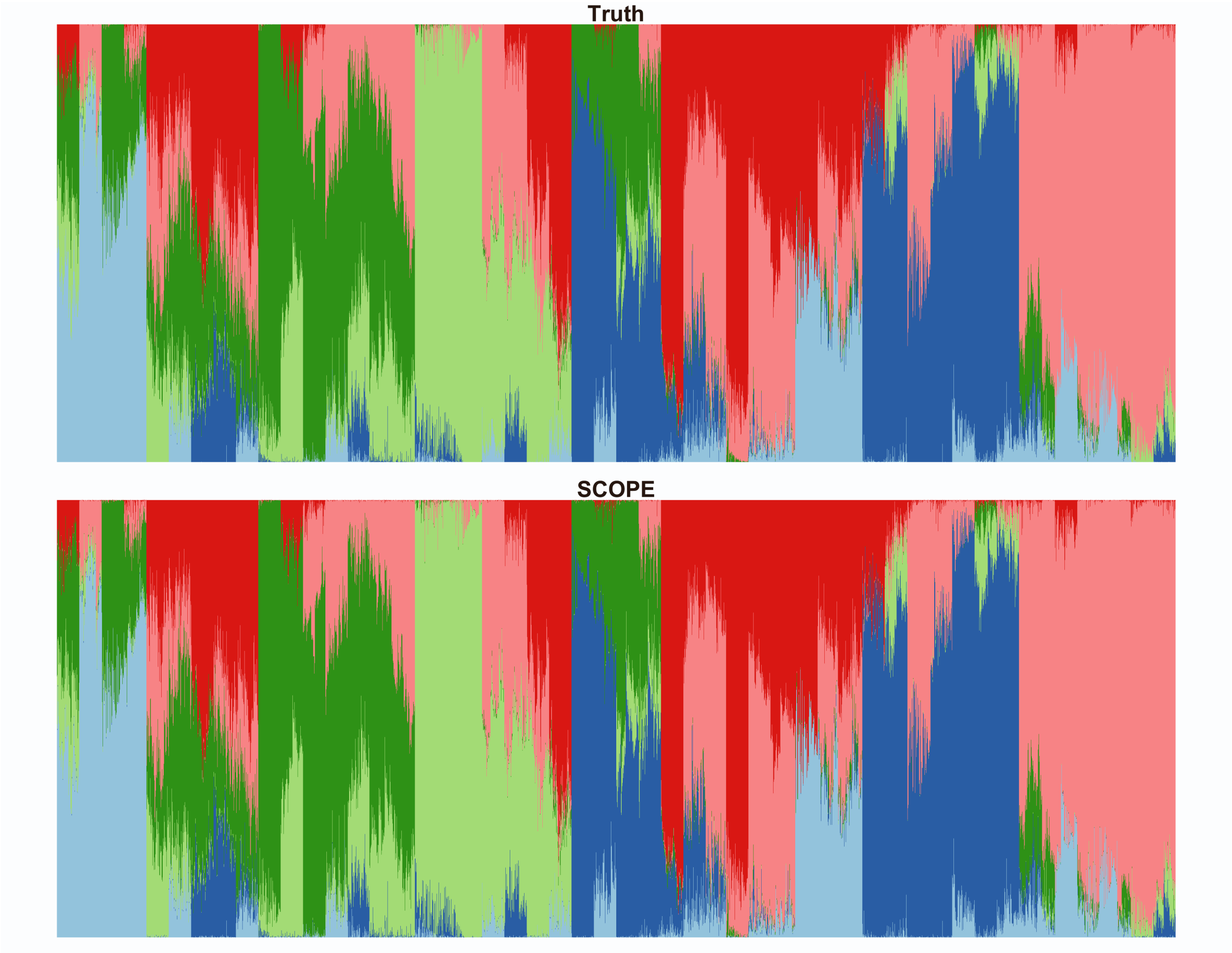

Figure S16: **Supervised population structure inference for simulations under the PSD model generated using 1000 Genomes Phase 3 data.** PSD model parameters were drawn from TGP data to generate a simulation dataset with 1 million individuals SNPs. SCOPE was provided the true population allele frequencies as input. Colors and order of samples are matched between SCOPE and the truth.

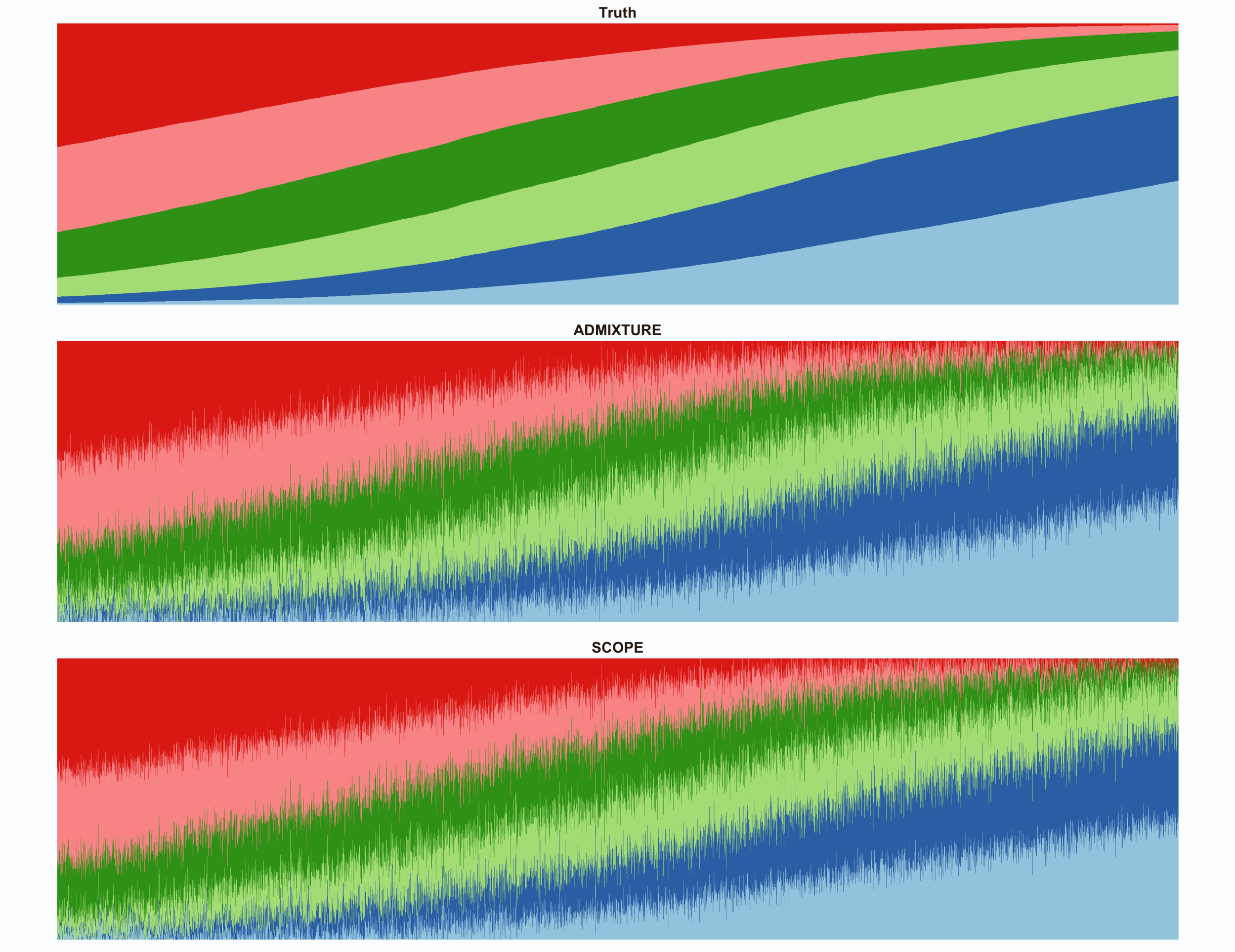

Figure S17: **Supervised population structure inference for simulations under a spatial model generated using Human Genome Diversity Project data.** Model parameters were drawn from HGDP data to generate a simulation dataset with 10,000 samples and 10,000 SNPs under a spatial model. Both methods were provided the true population allele frequencies as input. Colors and order of samples are matched between each method to the truth.

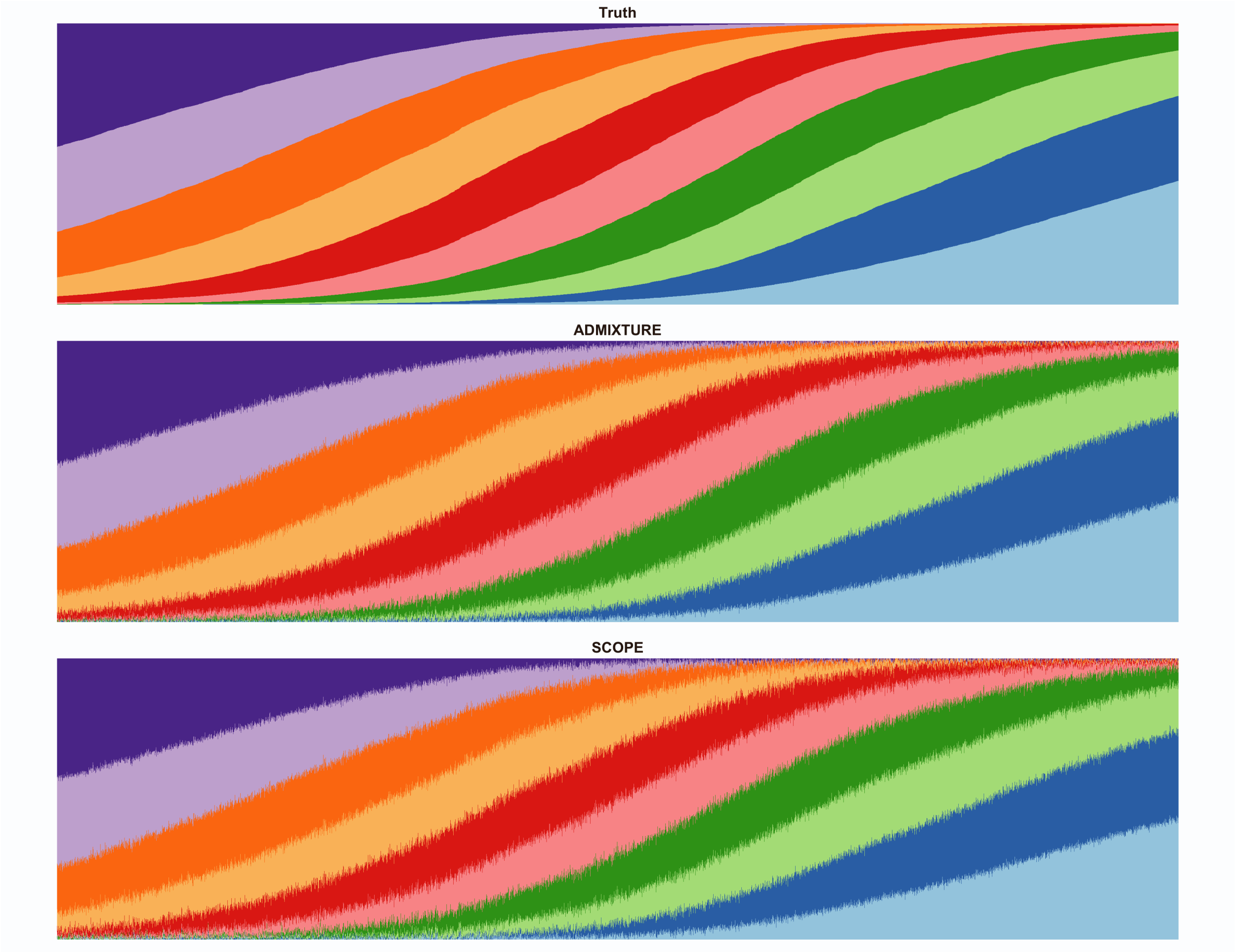

Figure S18: **Supervised population structure inference for simulations under a spatial model generated using 1000 Genomes Phase 3 data.** Model parameters were drawn from TGP data to generate a simulation dataset with 10,000 samples and 100,000 SNPs under a spatial model. Both methods were provided the true population allele frequencies as input. Colors and order of samples are matched between each method to the truth.

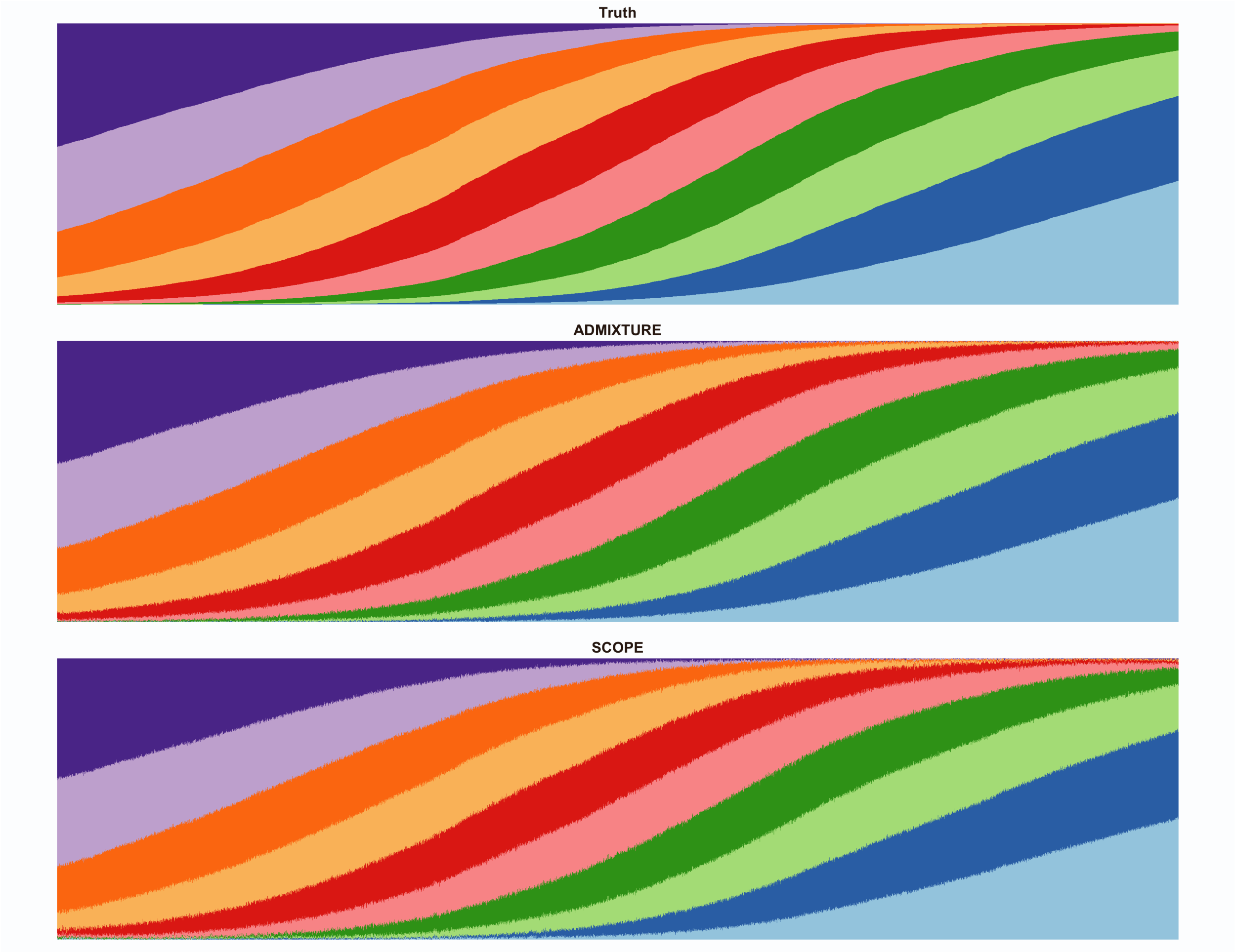

Figure S19: **Supervised population structure inference for simulations under a spatial model generated using 1000 Genomes Phase 3 data.** Model parameters were drawn from TGP data to generate a simulation dataset with 10,000 samples and 1 million SNPs under a spatial model. Both methods were provided the true population allele frequencies as input. Colors and order of samples are matched between each method to the truth.

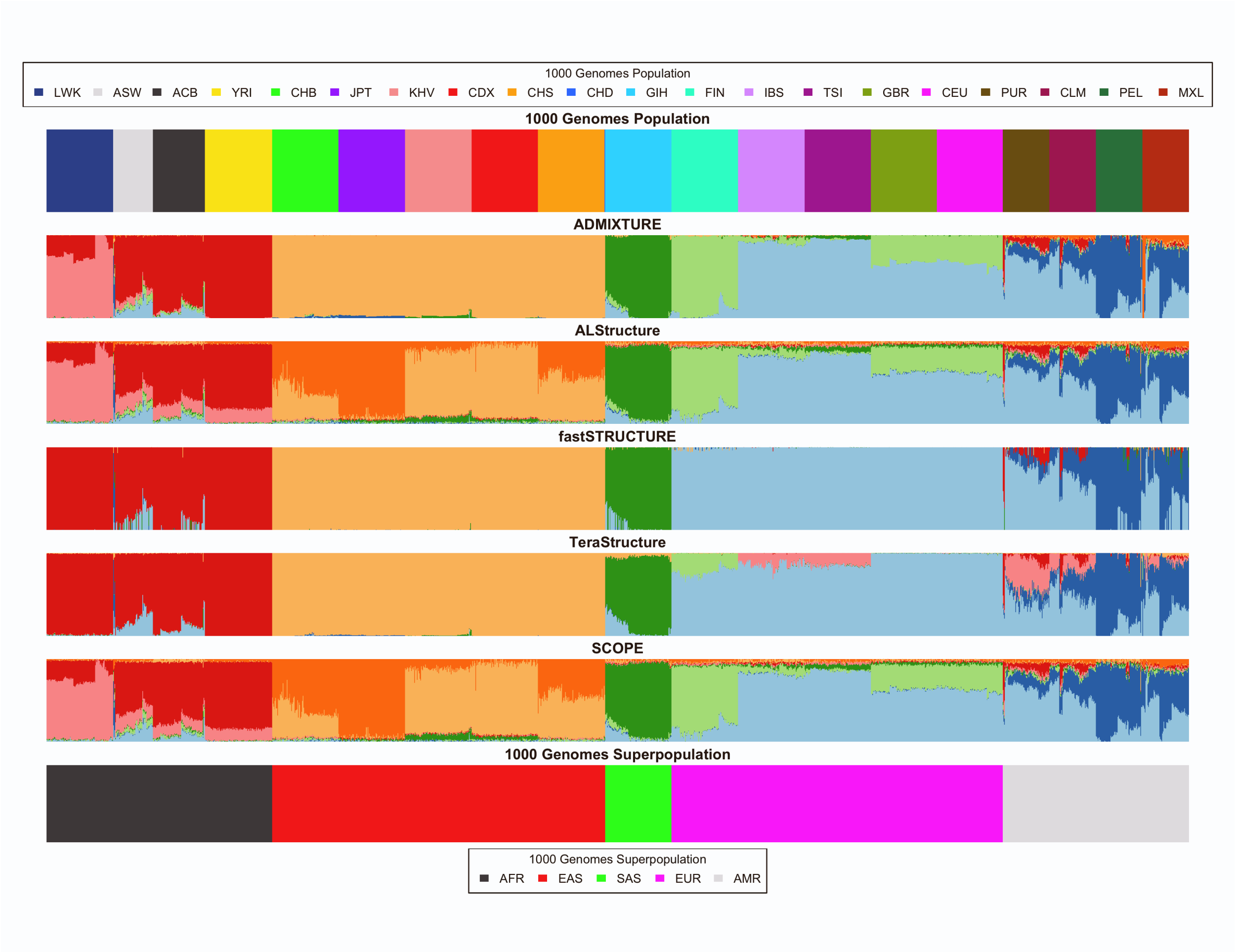

Figure S20: **Population structure inference of 1000 Genomes Phase 3 data using 8 latent populations.** Colors are matched between each method and ADMIXTURE. Samples are ordered through hierarchical clustering (see Methods). The superpopulations and superpopulations are shown for reference.

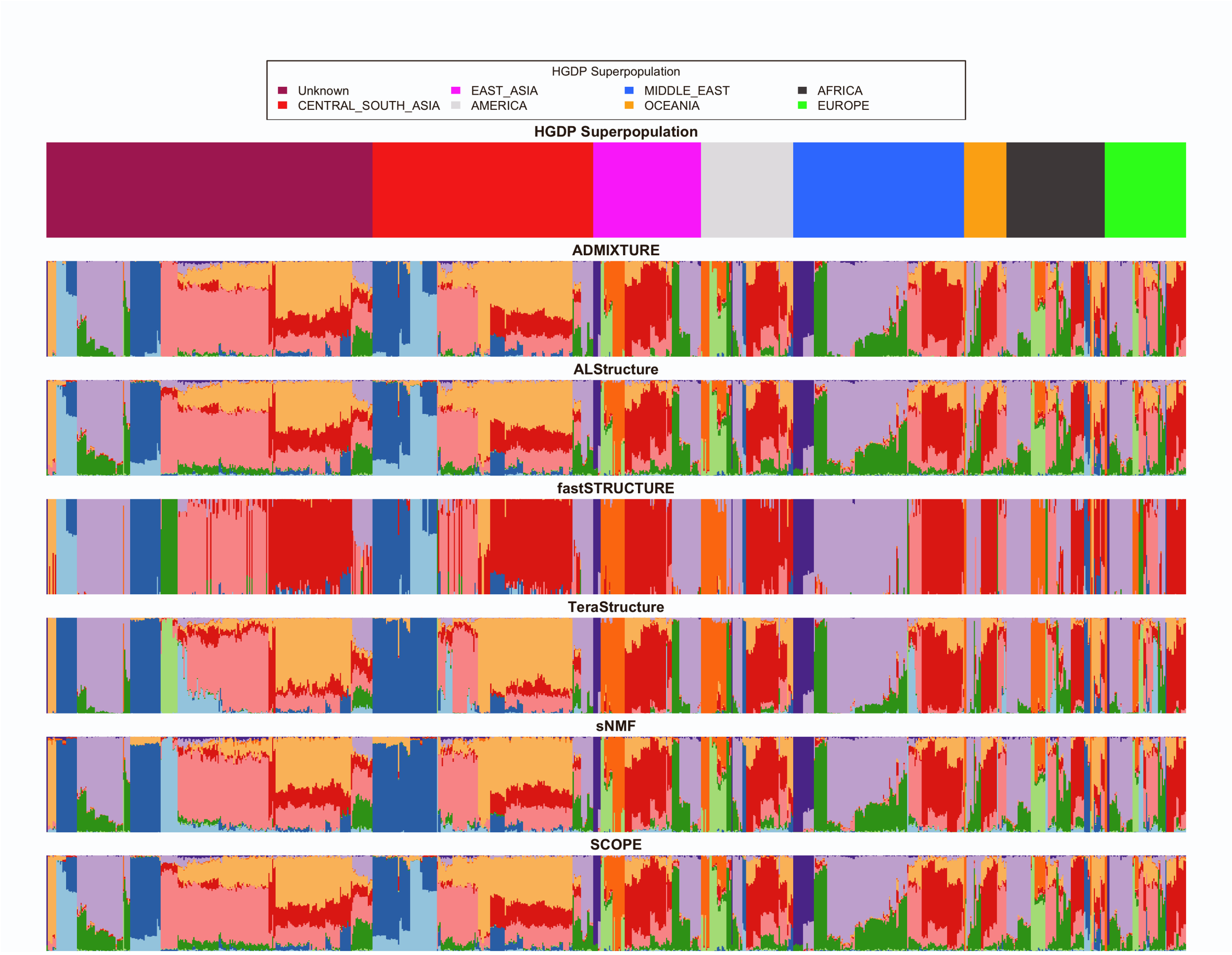

Figure S21: **Population structure inference of Human Genomes Diversity Population data using 10 latent populations.** Colors are matched between each method and ADMIXTURE. Samples are ordered through hierarchical clustering (see Methods). HGDP superpopulation is shown for reference.

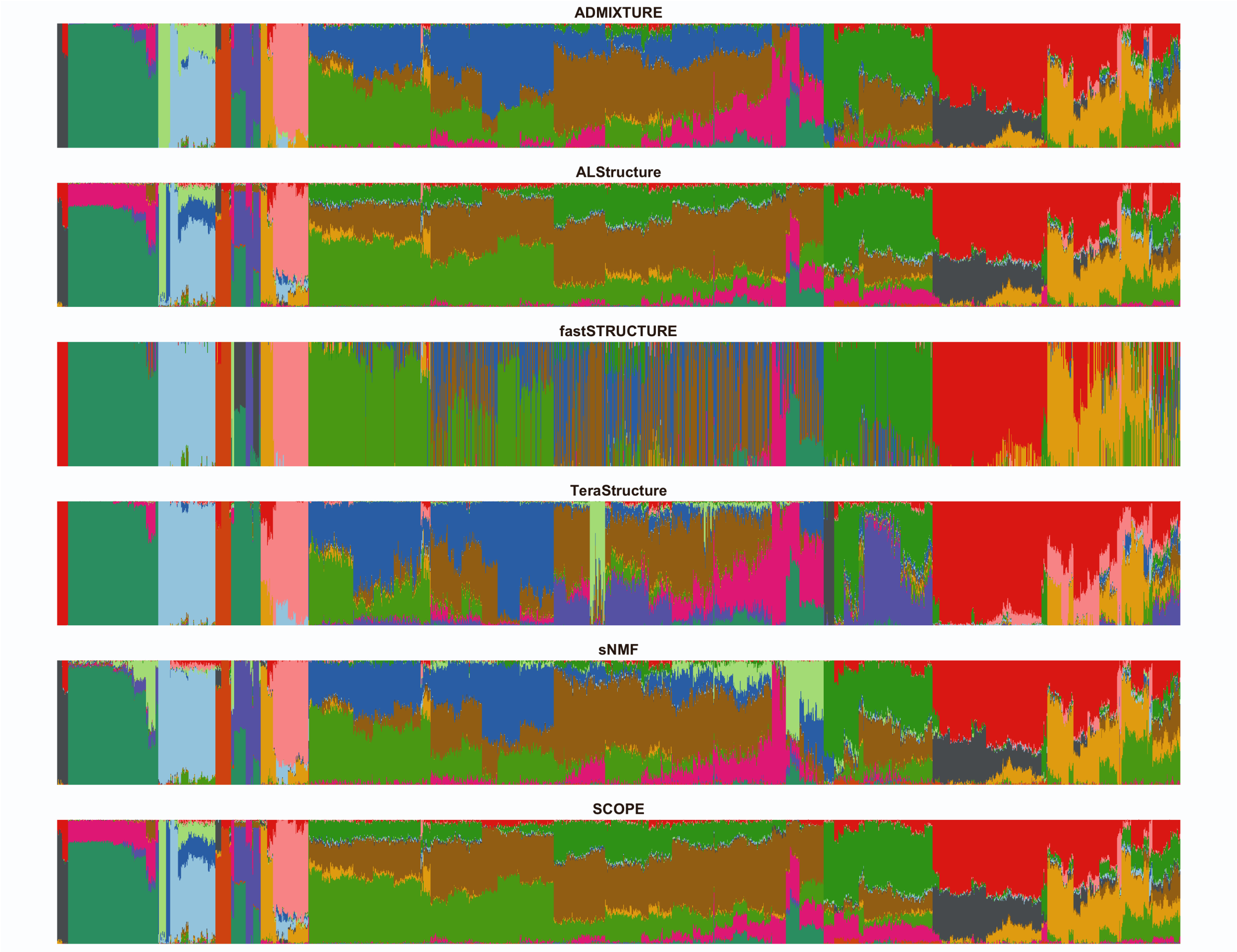

Figure S22: **Population structure inference of Human Origins data using 14 latent populations.** Colors and order of samples are matched between each method and ADMIXTURE. ADMIXTURE was ordered through hierarchical clustering (see Methods).

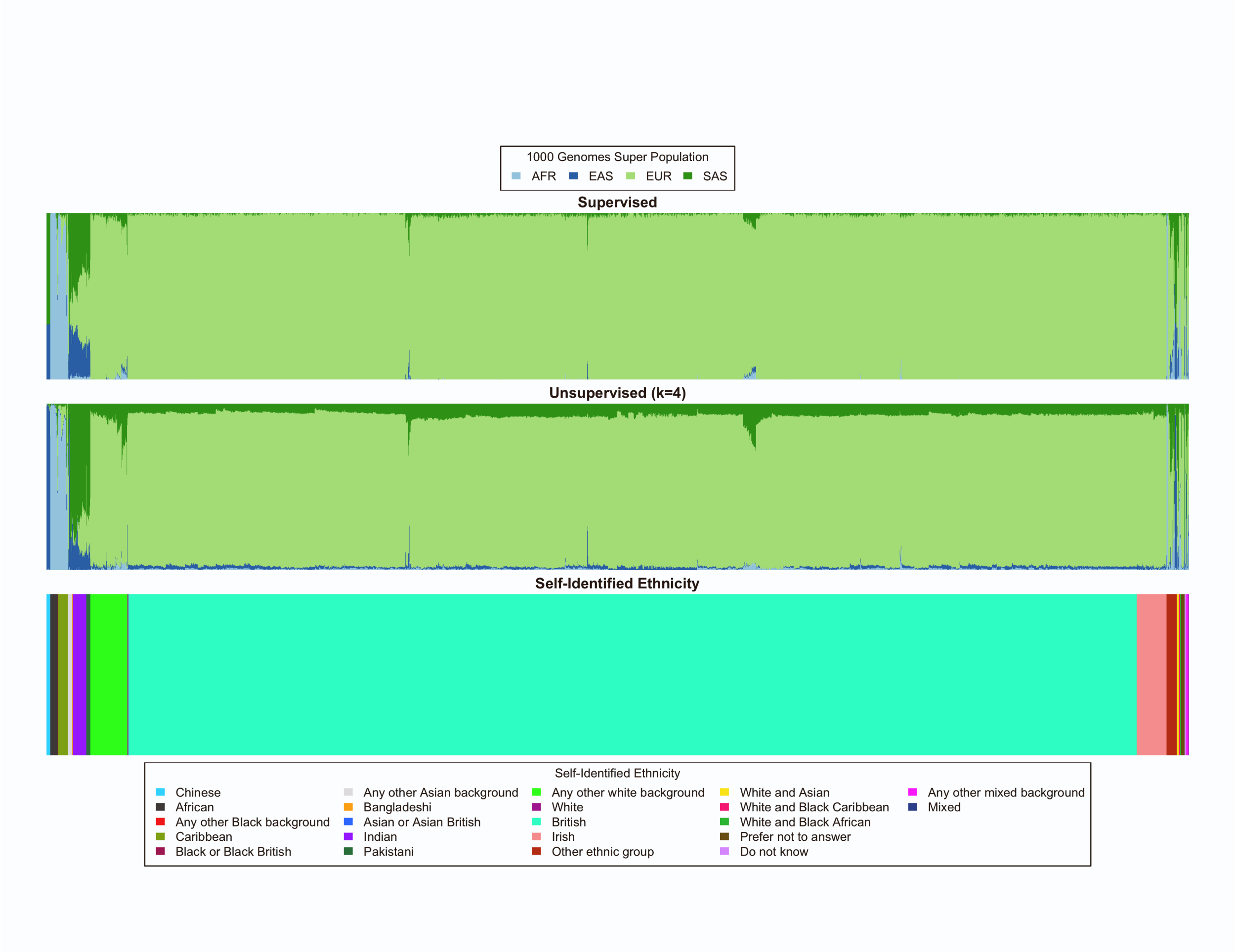

Figure S23: **Population structure inference on the UK Biobank with all individuals.** We ran population structure inference using SCOPE (488,363 individuals and 569,346 SNPs) in both supervised mode using 1000 Genomes Phase 3 allele frequencies (top) and unsupervised with 4 latent populations (middle). For reference, we plot the self-identified race/ethnicity (bottom). Colors and order of samples are matched between each row of the figure. This is an extended version of Figure 4 that includes all self-identified British samples.

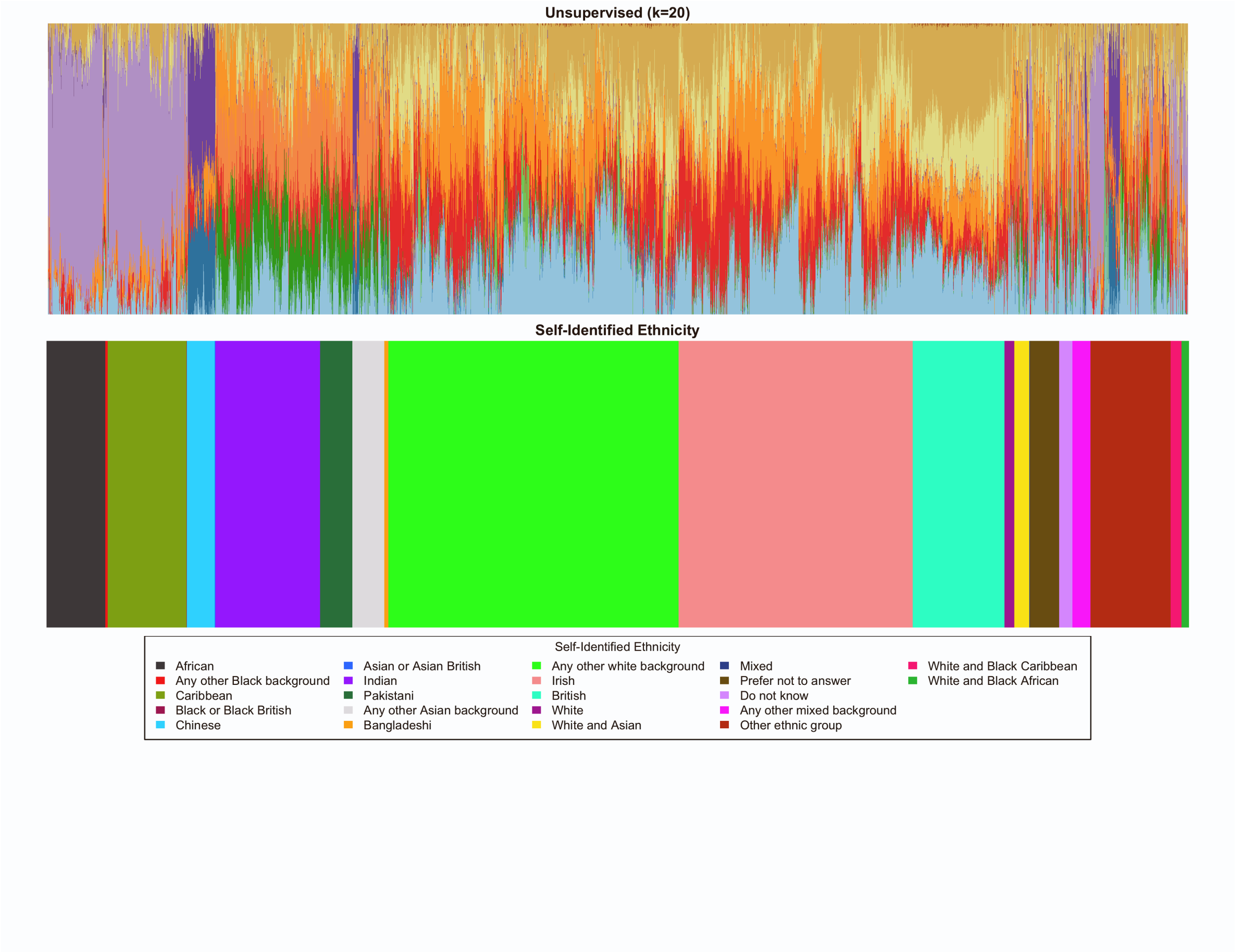

Figure S24: **Population structure inference on the UK Biobank with 20 latent populations.** We ran population structure inference using SCOPE unsupervised with 20 latent populations on the UK Biobank (488,363 individuals and 147,604 SNPs) (top). For reference, we plot the self-identified race/ethnicity (bottom). For visualization purposes, we reduced the number of self-identified British individuals to a random subset of 5,000 individuals. Colors and order of samples are matched between each row of the figure.

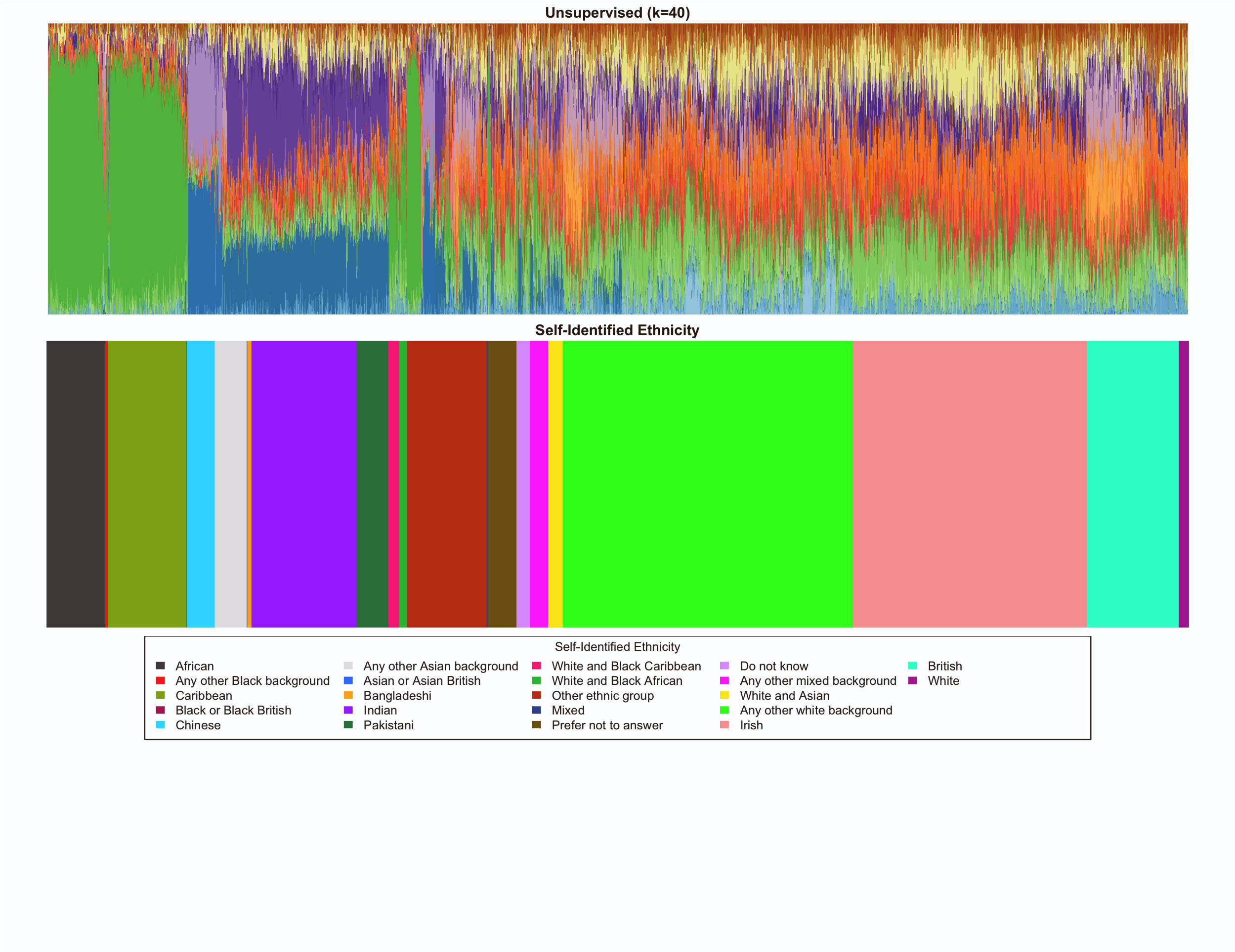

Figure S25: **Population structure inference on the UK Biobank with 40 latent populations.** We ran population structure inference using SCOPE unsupervised with 40 latent populations on the UK Biobank (488,363 individuals and 147,604 SNPs) (top). For reference, we plot the self-identified race/ethnicity (bottom). For visualization purposes, we reduced the number of self-identified British individuals to a random subset of 5,000 individuals. Colors and order of samples are matched between each row of the figure.

Table S1: **Kullback-Leibler divergence measurements for methods on simulated data with truth as first input.** Kullback-Leibler divergence (KLD) was computed against the ground truth admixture proportions for each simulation using truth as first input. Values are displayed as percentages rounded to one decimal place. Estimated proportions of 0 were set to 1×10^{-9} (see Methods). A '-' denotes that the method was not run due to projected time or memory usage. Bold values denote the best value for each dataset.

Dataset Type	Base Dataset	k	n	m	ADMIXTURE	fastStructure	TeraStructure	ALStructure	sNMF	SCOPE
PSD	HGDP	6	10,000	10,000	**8.3**	124.6	48.4	12.3	8.8	12.3
PSD	TGP	6	10,000	10,000	**3.4**	233.5	35.5	7.1	8.8	7.1
PSD	TGP	6	10,000	1,000,000	**0.2**	320.8	0.9	-	-	0.5
PSD	TGP	6	100,000	1,000,000	-	-	1.1	-	-	**0.6**
PSD	TGP	6	1,000,000	1,000,000	-	-	-	-	-	**0.7**
Spatial	HGDP	6	10,000	10,000	49.22	630.6	20.9	25.6	**15.3**	31.5
Spatial	TGP	6	10,000	10,000	62.8	596.7	**9.25**	60.6	25.7	58.6
Spatial	TGP	10	10,000	100,000	134.0	778.1	**27.2**	116.9	47.91	85.2
Spatial	TGP	10	10,000	1,000,000	-	-	**30.5**	-	-	85.6

Table S2: **Kullback-Leibler divergence measurements for methods on simulated data with truth as second input.** Kullback-Leibler (KLD) was computed against the ground truth admixture proportions for each simulation using truth as second input. Values are displayed as percentages rounded to one decimal place. Estimated proportions of 0 were set to 1×10^{-9} (see Methods). A '-' denotes that the method was not run due to projected time or memory usage. Bold values denote the best value for each dataset.

Dataset Type	Base Dataset	k	n	m	ADMIXTURE	fastStructure	TeraStructure	ALStructure	sNMF	SCOPE
PSD	HGDP	6	10,000	10,000	313.5	**219.8**	1560.7	476.9	311.5	476.0
PSD	TGP	6	10,000	10,000	**91.84**	197.9	769.7	260.8	311.5	259.3
PSD	TGP	6	10,000	1,000,000	**1.6**	175.9	16.0	-	-	25.87
PSD	TGP	6	100,000	1,000,000	-	-	40.4	-	-	**35.6**
PSD	TGP	6	1,000,000	1,000,000	-	-	-	-	-	**38.3**
Spatial	HGDP	6	10,000	10,000	24.9	127.2	30.4	**8.0**	8.8	9.9
Spatial	TGP	6	10,000	10,000	25.1	111.0	**10.8**	12.3	15.0	11.8
Spatial	TGP	10	10,000	100,000	56.8	136.8	33.7	32.3	23.9	**22.9**
Spatial	TGP	10	10,000	1,000,000	-	-	**29.2**	-	-	29.5

Table S3: **Memory usage of methods on simulated and real datasets.** ADMIXTURE, TeraStructure, sNMF, and SCOPE were run using 8 threads. ALStructure and fastStructure were run on a single thread due to their lack of multithreading implementations. TeraStructure's '-rfreq' parameter was set to 10% of the number of SNPs. A '-' denotes that the method was not run due to projected time or memory usage. Default parameters were used otherwise. Memory is displayed in gigabytes (GB). Bold values denote the best value for each dataset.

Dataset Type	Base Dataset	k	n	m	ADMIXTURE	fastStructure	TeraStructure	ALStructure	sNMF	SCOPE
PSD	HGDP	6	10,000	10,000	0.12	0.17	0.12	7.30	**0.04**	0.14
PSD	TGP	6	10,000	10,000	0.12	0.16	0.12	7.30	**0.04**	0.14
PSD	TGP	6	10,000	1,000,000	10.66	10.66	**9.96**	-	-	12.60
PSD	TGP	6	100,000	1,000,000	-	-	94.38	-	-	**93.47**
PSD	TGP	6	1,000,000	1,000,000	-	-	-	-	-	**746.19**
Spatial	HGDP	6	10,000	10,000	0.12	0.17	0.12	7.30	**0.04**	0.14
Spatial	TGP	6	10,000	10,000	0.12	0.16	0.12	7.30	**0.04**	0.14
Spatial	TGP	10	10,000	100,000	1.17	1.33	1.05	33.20	**0.38**	1.28
Spatial	TGP	10	10,000	1,000,000	-	-	**10.30**	-	-	12.69
Real	HGDP	10	940	642,951	1.94	1.99	1.17	24.38	**0.36**	1.30
Real	HO	14	1,931	385,089	1.83	1.89	1.21	27.45	**0.38**	1.53
Real	TGP	8	1,718	1,854,622	6.20	6.18	**4.44**	145.49	-	6.34
Real	UKB	4	488,363	569,346	-	-	-	-	-	**230.57**
Real	UKB	20	488,363	147,604	-	-	-	-	-	**60.92**
Real	UKB	40	488,363	147,604	-	-	-	-	-	**62.01**

Table S4: **Accuracy of supervised population structure inference for SCOPE and ADMIXTURE using supplied allele frequencies on simulations.** True allele frequencies were supplied to each method. Root-mean-square error (RMSE) and Jensen-Shannon Divergence (JSD) were computed against the true admixture proportions. Estimated proportions of 0 were set to 1×10^{-9} for JSD calculations (see Methods). A "-" denotes that the method was not run for that dataset due to time or memory constraints. Values are displayed as percentages. Bold values denote the best value for each dataset.

					SCOPE		ADMIXTURE	
Dataset Type	Base Dataset	k	n	m	RMSE	JSD	RMSE	JSD
PSD	HGDP	6	10,000	10,000	2.9	1.5	**2.6**	**1.2**
PSD	TGP	6	10,000	10,000	2.0	0.9	**1.6**	**0.6**
PSD	TGP	6	10,000	1,000,000	**0.2**	0.1	**0.2**	**0.03**
PSD	TGP	6	100,000	1,000,000	**0.2**	0.1	**0.2**	**0.03**
PSD	TGP	6	1,000,000	1,000,000	**0.2**	**0.1**	-	-
Spatial	HGDP	6	10,000	10,000	**2.4**	**0.6**	3.2	0.9
Spatial	TGP	6	10,000	10,000	**1.7**	**0.3**	2.2	0.4
Spatial	TGP	10	10,000	100,000	**0.6**	**0.3**	0.7	**0.3**
Spatial	TGP	10	10,000	1,000,000	0.3	**0.1**	**0.2**	**0.1**

Table S5: **Prediction accuracy of self-identified race and ethnicity using inferred admixture proportions.** We trained multinomial logistic regression models using the inferred admixture proportions from each method to predict SIRE labels. For TGP, we predicted 5 superpopulation labels corresponding to continental ancestry from 8 inferred latent populations. For HGDP, we predicted 7 continental ancestry populations from 10 inferred latent populations. Training accuracy as a percentage is reported. sNMF was not able to be run on TGP due to its disk space requirements.

Method	TGP	HGDP
ADMIXTURE	100	46.4
ALStructure	100	47.6
fastStructure	99.4	41.8
TeraStructure	100	47.8
sNMF	-	47.6
SCOPE	100	47.2

Table S6: **Prediction accuracy of birth location GPS coordinates for British individuals in the UK Biobank.** We trained ordinary least squares models using admixture proportions inferred by SCOPE from the three different runs on the UK Biobank. Two separate models were trained to predict the longitude coordinate and latitude coordinate. Quantiles of the difference between predicted birth location and reported birth location are displayed after the two R^2 columns and are reported in kilometers.

Number of Latent Populations	R^2 (Latitude)	R^2 (Longitude)	Minimum	25%	50%	75%	90%	95%	99%	Maximum
4	0.007	0.008	0.989	66.859	159.390	211.687	287.527	336.069	382.546	854.593
20	0.300	0.150	0.028	60.358	108.489	181.209	241.689	292.441	386.268	892.224
40	0.230	0.149	0.079	63.429	117.495	189.312	252.232	297.463	392.643	871.836
